# A consistent arrhythmogenic trait in Brugada syndrome cellular phenotype

**DOI:** 10.1002/ctm2.413

**Published:** 2021-06-06

**Authors:** Zeina R. Al Sayed, Mariam Jouni, Jean‐Baptiste Gourraud, Nadjet Belbachir, Julien Barc, Aurore Girardeau, Virginie Forest, Aude Derevier, Anne Gaignerie, Caroline Chariau, Bastien Cimarosti, Robin Canac, Pierre Olchesqui, Eric Charpentier, Jean‐Jacques Schott, Richard Redon, Isabelle Baró, Vincent Probst, Flavien Charpentier, Gildas Loussouarn, Kazem Zibara, Guillaume Lamirault, Patricia Lemarchand, Nathalie Gaborit

**Affiliations:** ^1^ l'institut du thorax Inserm CNRS UNIV Nantes Nantes France; ^2^ l'institut du thorax CHU Nantes Nantes France; ^3^ Nantes Université CHU Nantes, Inserm CNRS SFR Santé Nantes France; ^4^ Laboratory of Stem Cells PRASE Biology Department Faculty of Sciences Lebanese University Beirut Lebanon

To the Editor:

Brugada syndrome (BrS) is an inherited arrhythmic disease predisposing to sudden cardiac death (SCD), characterized by a typical electrocardiogram pattern that includes a J point elevation with a coved type ST segment.^1^ BrS is a complex genetic disease in which ∼20% of patients carry rare variants in *SCN5A* gene, whereas the others remain genetically unresolved.^2^ Despite this genetic complexity, we hypothesize that a common cellular phenotypic trait is at the root of this specific BrS ECG pattern. In this study, we identified a phenotype that is common to human‐induced pluripotent stem cell‐derived ventricular cardiomyocytes (hiPSC‐CMs) generated from six Brugada patients with different genetic backgrounds. Our results unmasked a cellular arrhythmogenic phenotype combining gene expression and electrical abnormalities, including an increase in late sodium current.

Six patients affected by type I BrS (BrS1‐6; Figure S1; Tables S1 and S2) with a familial history of SCD or syncope were selected, among whom two carry *SCN5A* variants (marked with a ^+^ symbol). An additional individual, not affected by BrS (non‐BrS), carrying the same *SCN5A* variant as BrS2^+^, was also recruited, as well as four control (Ctrl) subjects. Somatic cells from all studied subjects were reprogrammed into hiPSC lines and differentiated into cardiomyocytes (Figure 1).

**FIGURE 1:**
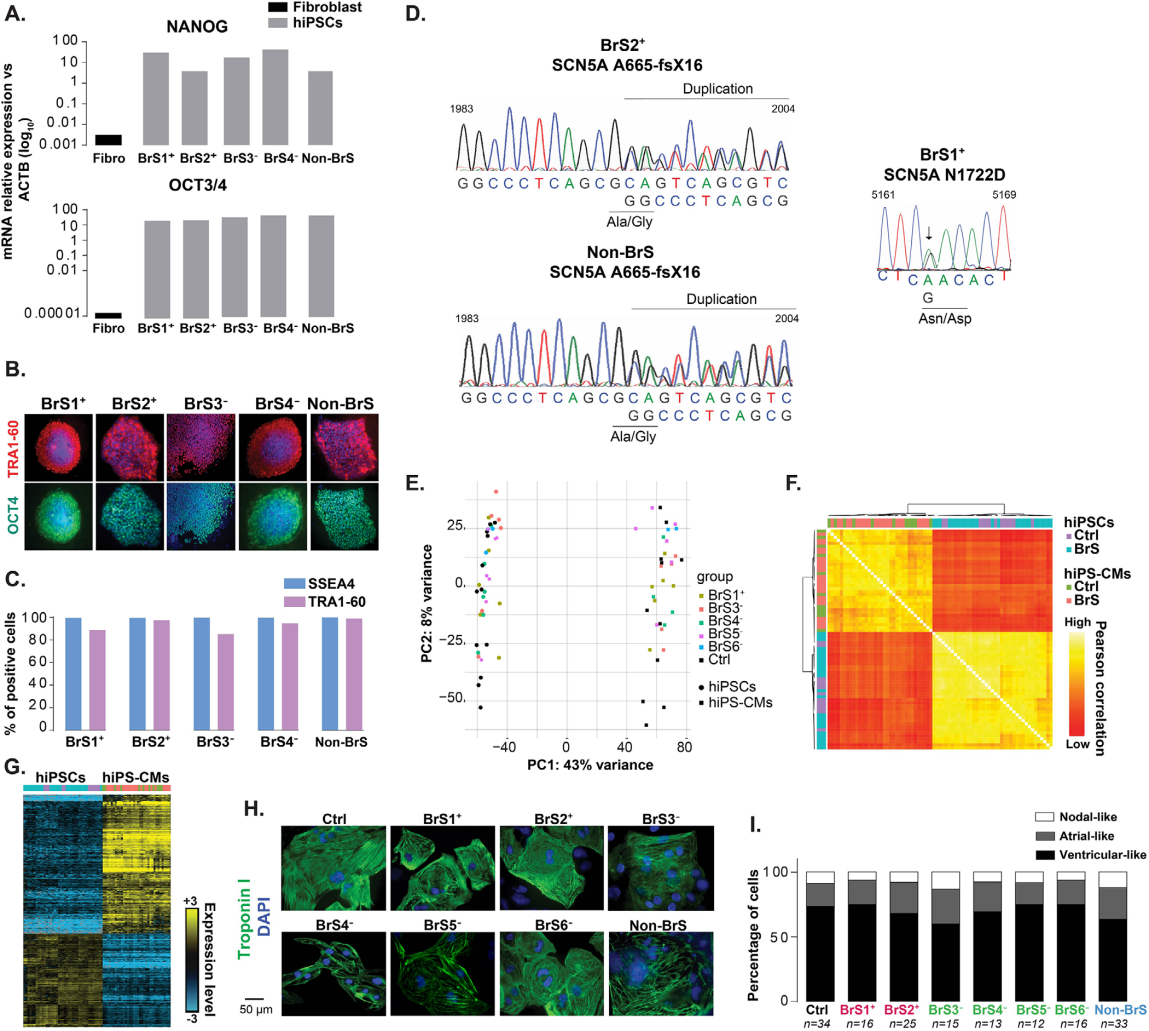
Pluripotency and *SCN5A* variant validation in hiPSCs, and characterization of derived cardiomyocytes. (A) Transcript expression of pluripotency markers: NANOG and OCT3/4 in newly described hiPSCs as compared to fibroblasts (Fibro). (B) Representative immunostainings of TRA1‐60 (red) and OCT4 (green) in hiPSCs. (C) Percentage of hiPSCs expressing SSEA4 and TRA1‐60 evaluated by flow cytometry. (D) Genomic sequence chromatograms validating (right) the 5164A>G *SCN5A* variant carried by BrS1^+^ and (left) the *SCN5A* 1983‐1993 duplication carried by BrS2^+^ and non‐BrS, in the corresponding hiPSCs. (E) Principal component analysis (PCA) of 39 hiPSC samples and their corresponding differentiated hiPSC‐CMs, based on their expression pattern of 27106 analyzed transcripts (3′SRP data). All clones of each hiPSC line are highlighted. (F) Correlation matrix of hiPSCs and hiPSC‐CMs expression profiles. Yellow and orange indicate high and low correlation, respectively. Samples were clustered using an ascending hierarchical method with Pearson as metric and ward D2 linkage. (G) Heatmap showing expression levels of 9661 differentially expressed genes between hiPSCs and hiPSC‐CMs (same samples as in A). Genes were clustered using a hierarchical ascending method with an uncentered correlation metric and complete linkage. Yellow and blue indicate high and low levels, respectively. (H) Illustrative immunostainings of Troponin I (green) in hiPSC‐CMs. Nuclei were stained with DAPI (blue). (I) Percentages of nodal‐like, atrial‐like, and ventricular‐like cells classified based on the analysis of spontaneous action potential recordings

Transcriptional expression profiling identified 133 differentially expressed genes in BrS hiPSC‐CMs (Figure 2A). gene set enrichment analyses showed that transcripts of transmembrane transporters and channels were significantly overrepresented (Figure 2B), including genes encoding sodium, calcium, and potassium channels (Figure 2C). High‐throughput real‐time RT‐PCR^3^ on 96 genes related to cardiac electrical function (Table S3) identified 13 differentially expressed genes in BrS, in comparison to Ctrl and non‐BrS hiPSC‐CMs (Figure 2D). Importantly, the expression of *SCN5A*, the main BrS culprit gene identified to date,^4^ remained unchanged, excluding *SCN5A* expression levels as a hallmark for BrS hiPSC‐CM phenotype. Conversely, calcium and sodium transporters, playing important roles in membrane depolarization, were differentially expressed. Comparative analysis of hiPSC‐CM electrophysiological functions investigated whether these modifications were a consistent trait of BrS phenotype at the cellular level.

**FIGURE 2:**
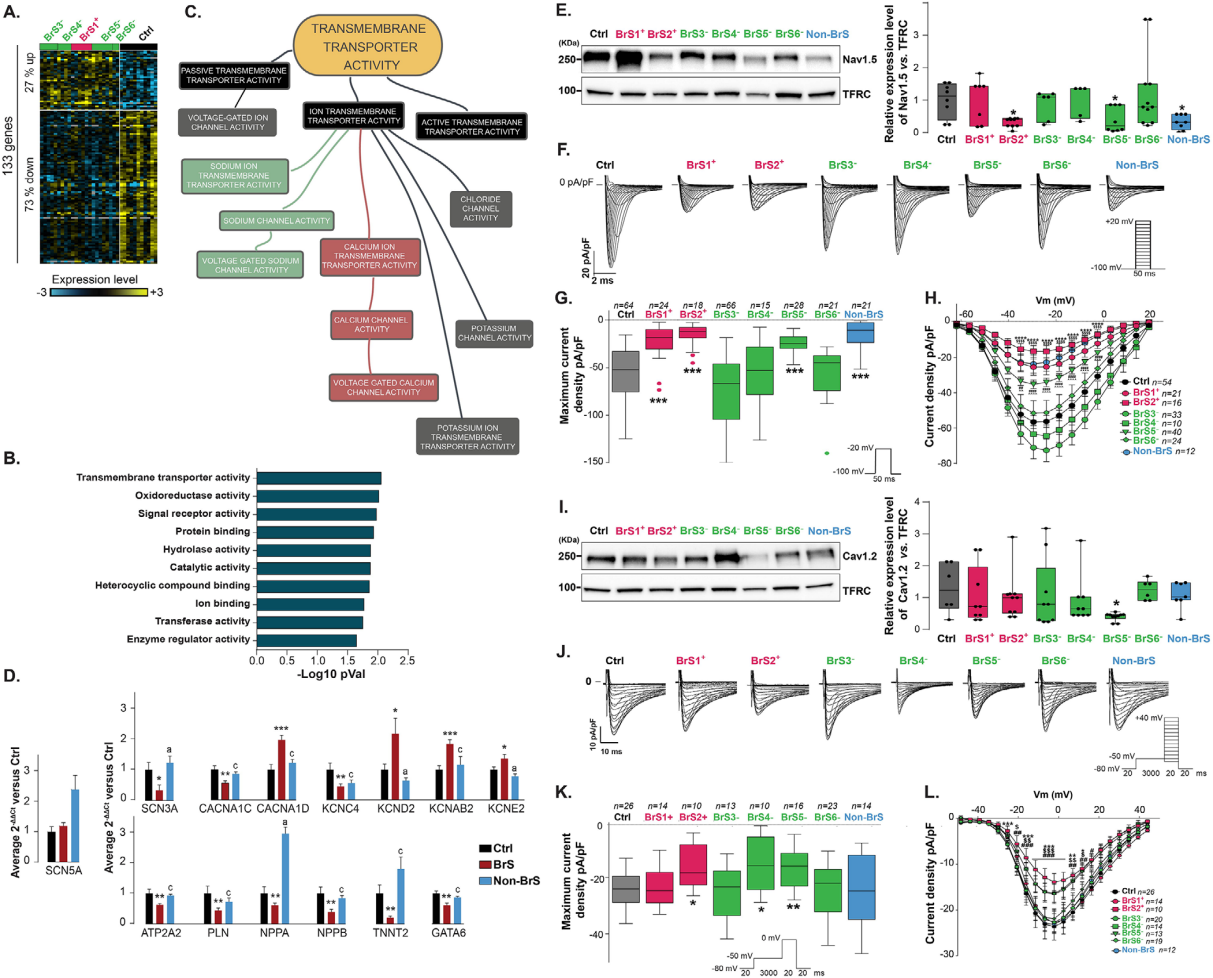
Differential gene expression profiles and variations in *I*_Na_ and *I*_Ca,L_, in BrS hiPSC‐CMs as compared to controls. (A) Heatmap showing hierarchical clustering of expression profiles of 133 differentially expressed genes obtained by 3′SRP in control (Ctrl) and BrS hiPSC‐CMs at day 28 of differentiation. A total of 27% were upregulated, whereas 73% genes were downregulated in BrS hiPSC‐CMs. Yellow and blue represent high and low expression levels, respectively. All clones of each hiPSC line are highlighted. (B) Gene set enrichment analysis (GSEA) of gene variations obtained by 3′SRP shows gene sets with statistically altered expression patterns. (C) MindMap describing the transmembrane transporter activity alterations. (D) Expression levels of differentially expressed genes identified using high‐throughput TaqMan (TLDA) in BrS hiPSC‐CMs (*n* = 14), compared to control hiPSC‐CMs (*n* = 12), and in non‐BrS hiPSC‐CMs (*n* = 4) versus BrS hiPSC‐CMs. *p*‐values: *, **, and *** or a, b, and c: *p* < .05, *p* < .01, and *p* < .001 versus Ctrl or BrS, respectively (*t*‐test). (E) Representative immunoblots for Na_v_1.5 and transferrin receptor (TFRC) in hiPSC‐CMs (left panel). Ratios of Na_v_1.5 expression levels (right panel, Tukey plot, *n* = 8). *p < .05 versus control (Mann–Whitney test). Na_v_1.5 decreases in hiPSC‐CMs from three subjects, BrS2^+^ and non‐BrS (both carrying a stop codon in *SCN5A*), as well as BrS5^−^, harboring *RRAD* variant was observed. (F) Representative superimposed *I*_Na_ densities (inset: voltage‐clamp protocol). Reduction was detected in BrS2^+^, BrS5^−^, and non‐BrS, as well as in BrS1^+^ hiPSC‐CMs carrying the N1722D‐*SCN5A* rare variant. (G) Peak *I*_Na_ densities measured in control (Ctrl), BrS, and the non‐affected carrier of *SCN5A* mutation (non‐BrS) hiPSC‐CMs determined at −20 mV (Tukey plot). ***p < .001 versus control (Mann–Whitney test). (H) Mean peak *I*_Na_ densities (pA/pF) versus membrane potential (*V*_m_) recorded in hiPSC‐CMs. ****, $$$$, ####, and ^^^^ *p* < .0001 versus control for BrS1^+^, BrS2^+^, BrS5^−^, and non‐BrS, respectively (two‐way ANOVA with Bonferroni post hoc test). (I) Representative immunoblots for Ca_v_1.2, the main pore‐forming subunit of the cardiac L‐type calcium channel, and transferrin receptor (TFRC) in hiPSC‐CMs (left panel). Ratios of Ca_v_1.2 expression levels (right panel, Tukey plot, *n* = 8). A decrease in Ca_v_1.2 expression was solely observed in BrS5^−^ hiPSC‐CMs, carrying an *RRAD*‐variant. *p < .05 versus control (Mann–Whitney test). (J) Representative superimposed *I*_Ca,L_ densities (inset: voltage protocol). (K) Peak *I*_Ca,L_ densities measured in control (Ctrl), BrS, and the nonaffected carrier of *SCN5A* mutation (non‐BrS) hiPSC‐∖CMs determined at 0 mV (Tukey plot). A decrease in *I*_Ca,L_ was observed in BrS2^+^, BrS4^−^ and, consistently with a previous description, in BrS5^−^.^7^ *p < .05 and **p < .01 versus control (Mann–Whitney test). (L) Mean peak *I*_Ca,L_ densities (pA/pF) versus membrane potential (*V*_m_) recorded in hiPSC‐CMs. *, #, and $ *p* < .05, **, ##, and $$ *p* < .01, and ***, ###, and $$$ *p* < .001 versus control for BrS1^+^, BrS4^−^, and BrS5^−^, respectively (two‐way ANOVA with Bonferroni post hoc test)

Whereas decrease in sodium current is considered as the most frequently associated electrical alteration in BrS pathophysiology,^5^, ^6^ protein expression of Na_v_1.5, encoded by *SCN5A*, was decreased in only two BrS, and the non‐BrS lines (Figure 2E). Concordantly, reduction in *I*_Na_ density was detected in these same lines (Figure 2F–H). This confirmed previous results, for BrS5^+^,^7^ and regarding BrS1^+^, which carries an *SCN5A* rare variant, the reduction was confirmed using conventional transfection in COS‐7 cells of this variant (Figure S2). Furthermore, the steady‐state activation and inactivation gating properties were not modified in BrS hiPSC‐CMs (Figure S3A; Table S4). Therefore, *I*_Na_ reduction is not a common trait of BrS hiPSC‐CMs and appears to be solely associated with the presence of variants affecting *SCN5A* expression or function.

Similarly, reduction in *I*_Ca,L_ channel protein expression and current density were not a common trait of BrS hiPSC‐CMs (Figure 2I–L, Figure S3B; Table S4).

Global cellular electrophysiological phenotype was then evaluated with action potential (AP) recordings, but no AP basal parameters specifically segregated BrS hiPSC‐CMs, and spontaneous beating frequencies did not differ between all cell lines (Figure S4). Noteworthy, ventricular‐like AP analysis revealed an arrhythmic phenotype present mostly in BrS hiPSC‐CMs, irrespective of their genetic background (Figure 3A). Early afterdepolarizations (EADs) were observed in 39–70% of all six BrS ventricular‐like hiPSC‐CMs versus only in 4% and 4.7% of Ctrl and non‐BrS hiPSC‐CMs, respectively (Figure 3B, Figure S5). Thereby, the high EAD occurrence in ventricular‐like hiPSC‐CMs was associated with the presence of a BrS phenotype in the investigated cell lines, but not with the presence of a variant in *SCN5A*.

**FIGURE 3:**
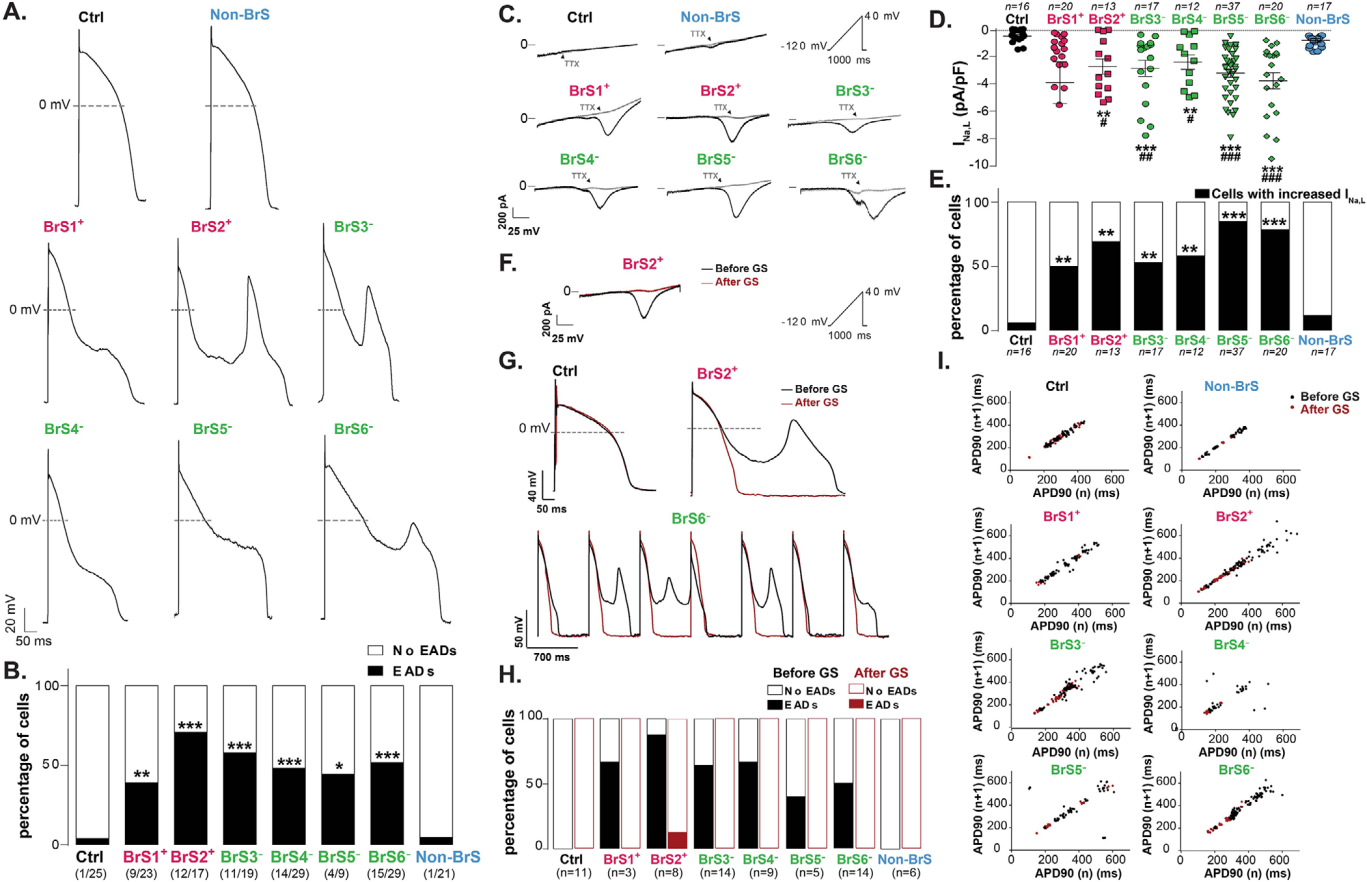
Increased early after depolarization (EAD) occurrence in all BrS ventricular‐like hiPSC‐CM lines, linked to an increase in late sodium current. (A) Representative AP recordings, showing EADs in BrS lines only. Representative ventricular‐like AP when paced at 700 ms cycle length and when artificial *I*_K1_ was injected (dynamic current clamp). APs are defined as ventricular‐like when (APD_30_ − APD_40_)/(APD_70_ − APD_80_) > 1.45. (B) Percentage of ventricular‐like hiPSC‐CMs presenting at least 1 EAD, irrespective of the current clamp conditions. *p < .05, **p < .01, and ***p < .001 versus control (Fisher's exact test). (C) Representative *I*_Na,L_ recordings from hiPSC‐CMs, before (black) and after (grey) TTX application (inset: voltage protocol). (D) *I*_Na,L_ (TTX‐sensitive current) densities at −10 mV. *p < .05 and ***p < .001 versus Ctrl (Mann–Whitney test), and ^##^ *p* < .01 and ^###^ *p* < .001 versus non‐BrS (Mann–Whitney test). (E) Percentage of cells presenting *I*_Na,L_ density greater than the 97th percentile value of *I*_Na,L_ in the Ctrl hiPSC‐CMs. **p < .01 and ***p < .001 versus control (Fisher's exact test). Indeed, an increase in *I*_Na,L_ density was defined by values higher than 97th percentile of the Ctrl hiPSC‐CMs. (F) Representative example of *I*_Na,L_ current recorded in BrS hiPSC‐CMs before (black) and after (red) application with GS‐458967 (300 nM), a specific *I*_Na,L_ inhibitor (inset: voltage protocol). (G) Representative AP recordings from control and BrS hiPSC‐CMs obtained before and after GS‐458967 application. (H) Percentage of cells with EADs before and after GS‐458967 application. (I) Poincaré plots showing APD_90_ of each AP (*n* + 1) versus APD_90_ of its preceding one, before and after GS‐458967 application

The occurrence of EADs may be linked to an abnormally high density of depolarizing late sodium current (*I*_Na,L_) during APs repolarizing phase.^8^ Accordingly, BrS hiPSC‐CMs presented with a higher density of *I*_Na,L_ as compared to Ctrl and non‐BrS hiPSC‐CMs (Figure 3C,D). Moreover, an increase in *I*_Na,L_ density was observed only in 6% and 12% of Ctrl and non‐BrS hiPSC‐CMs respectively, in accordance with their low EAD occurrence, whereas increased *I*_Na,L_ density was present in 50–85% of all BrS ventricular‐like hiPSC‐CMs, reminiscent of the high EAD occurrence (Figure 3B,E). We then superfused ventricular‐like BrS hiPSC‐CMs during AP recording with GS‐458967 (6‐(4‐(trifluoromethoxy)phenyl)‐3‐(trifluoromethyl)‐[1,2,4]triazolo[4,3‐a]pyridine, which selectively blocks late sodium current),^9^ causing full inhibition of *I*_Na,L_ (Figure 3F), and found abolishment of EADs (Figure 3G,H) and reduced APD90 dispersion (Figure 3I). Altogether, these data strongly suggested that the abnormal increase of *I*_Na,L_ in BrS hiPSC‐CMs is responsible for EADs.

Further strengthening the role of *I*_Na,L_ in the electrical cellular phenotype of BrS, when each ECG parameter was tested for its correlation with either *I*_Na,L_ or *I*_Na_ measured densities, only *I*_Na,L_ density correlated significantly with one sole parameter, that is, the *J* point elevation (Table S5).

To challenge the pathophysiological relevance of the ion current alterations identified in non‐BrS and BrS2^+^ hiPSC‐CMs, we applied them to a mathematical human electrogram model that allows visualizing transmural‐like electrogram with a QRS‐like complex, a ST‐like segment, and a T‐like wave (Figure 4A).^10^ First, in accordance with BrS2^+^ patient's ECG, applying the alterations observed in peak *I*_Na_, *I*_Ca,L_, and in *I*_Na,L_ in BrS2^+^ hiPSC‐CMs was sufficient to induce prolongation of the QRS‐like complex, ST‐like segment elevation and widening, and T‐like wave inversion (Figure 4B). Then, sequential correction of each altered current in BrS2^+^ hiPSC‐CMs was made (BrS2^+^_corrected_). Correction of *I*_Na_ density led to QRS‐like complex normalization; correction of *I*_Ca,L_ density shortened duration of the ST‐like segment elevation and normalized the *T*‐like wave orientation; and correction of *I*_Na,L_ density led to reduction of ST‐like segment amplitude toward normalization (Figure 4C, left to right). Overall, these results strongly suggest that depolarizing current alterations can impact a multicellular electrogram model, mimicking BrS ECG phenotype.

**FIGURE 4:**
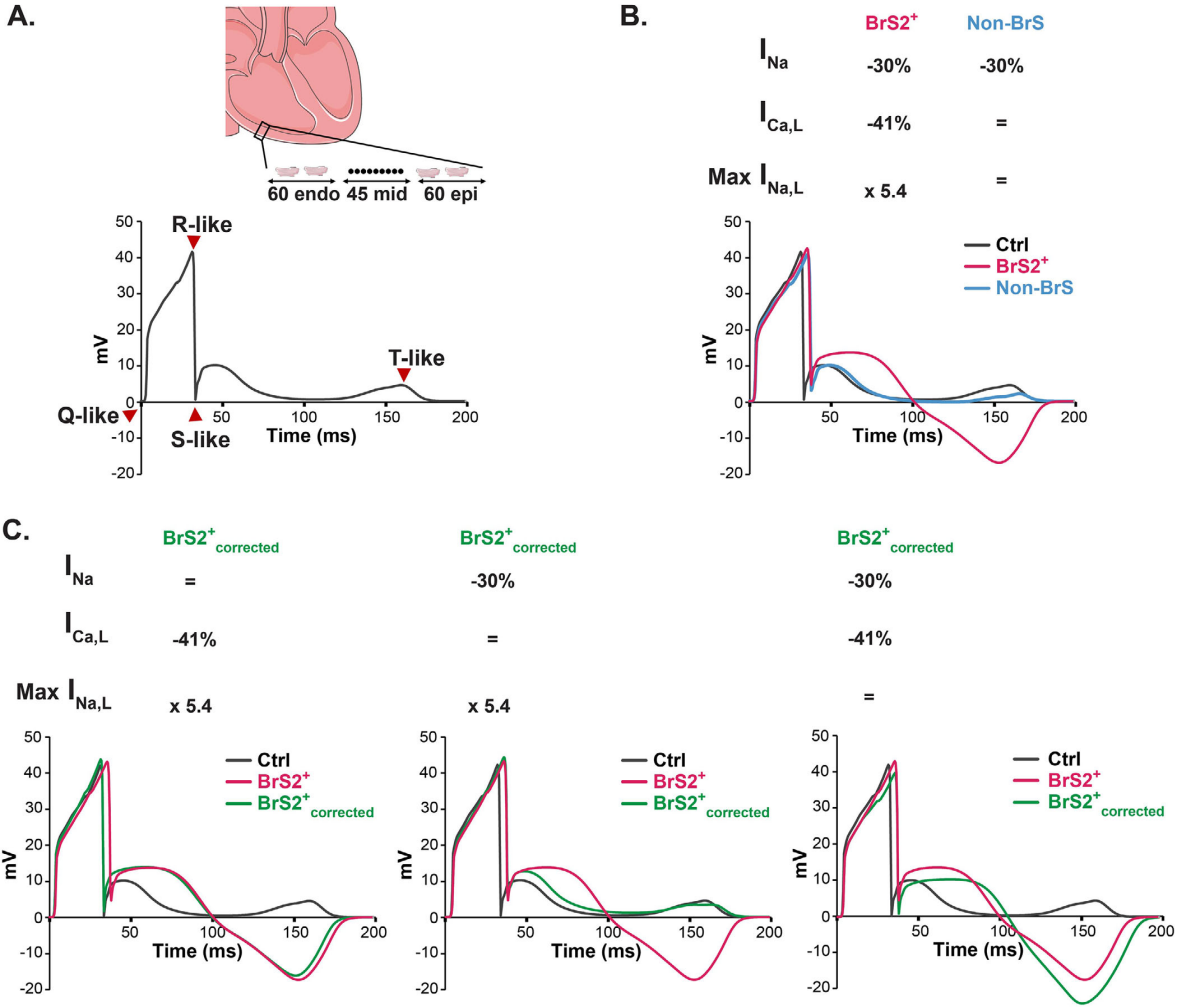
Applying depolarizing ion current alterations from BrS hiPSC‐CMs on an electrogram model to mimic BrS patient's ECG features. (A) Top: Right ventricle electrogram model simulates the global electrical activity of a transmural wedge comprising 60 subendocardial, 45 midmyocardial, and 60 subepicardial human ventricular cells. Bottom: Representative electrogram showing the Q‐like, R‐like, S‐like and T‐like waves. (B) Ventricular transmural electrogram mathematical model of Ctrl (black), BrS2^+^ (pink), and non‐BrS (blue) illustrated based on hiPSC‐CMs data of the relative variation in ion currents (*I*_Na_, *I*_Ca,L_, and *I*_Na,L_) mean amplitude as compared to Ctrl lines. In accordance with patient's ECGs, applying BrS2^+^ ionic current changes prolonged the QRS‐like complex, elevated and widened the ST‐like segment, and inversed the T‐like wave; and applying *I*_Na_ change identified in non‐BrS hiPSC‐CMs only prolonged the QRS‐like complex, similar to non‐BrS PCCD ECG. (C) Each of the currents *I*_Na_, *I*_Ca,L_, and *I*_Na,L_ (from left to right) were sequentially corrected and the resulting electrograms are illustrated in green. Ctrl and BrS2^+^ electrograms are presented in black and pink, respectively

In conclusion, in the present study, a particular cellular electrophysiological phenotype common to six out of six BrS hiPSC‐CM lines with various genetic backgrounds has been unveiled. We showed that high EAD occurrence associates with an abnormal increase of *I*_Na,L_ in all investigated BrS cell lines, and correlates with the corresponding patients’ *J* point elevation on ECG. We focused on the ventricular cell type, at a single‐cell level. Implementation of emerging phenotypic technologies, such as single‐cell transcriptomics and cardiac tissue engineering, will allow investigation of the potential involvement of other cardiac cell types in the disease phenotype and the role of specific cell‐to‐cell interactions. Altogether, the obtained results open perspectives to better understand the ventricular arrhythmia occurrence in BrS and to identify a dedicated therapeutic approach to prevent the risk of SCD.

## CONFLICT OF INTEREST

The authors declare that there is no conflict of interest.

## DATA AVAILABILITY STATEMENT

In accordance with the “DFG Guidelines on the Handling of Research Data,” the authors declare that all data supporting the findings of this study are available within the article and its supporting information files or from the corresponding author upon reasonable request. The dataset will be archived for at least 10 years after publication.

## FUNDING INFORMATION

Fondation pour la Recherche Médicale, Grant Number: DEQ20140329545; National Research Agency: Grant Number: ANR‐14‐CE10‐0001‐01 and HEART‐iPS Grant Number: ANR‐15‐CE14‐0019‐01; La Fédération Française de Cardiologie; Fondation Lefoulon‐Delalande; Marie Curie Actions: International Incoming Fellowship, Grant Number: FP7‐PEOPLE‐2012‐IIF (PIIF‐GA‐2012‐331436) and H2020‐MSCA‐IF‐2014 (RISTRAD‐661617); Eiffel Program of Excellence; Fondation Genavie; Association of Scientific Orientation and Specialization; Lebanese University; Data‐Santé (Région Pays de la Loire)

## Supporting information

Supporting Information
