# Supplementary material for: A consistent arrhythmogenic trait in Brugada syndrome cellular phenotype
Source: Clin Transl Med. 2021 Jun 6;11(6):e413. doi: 10.1002/ctm2.413 (PMC8181201; doi:10.1002/ctm2.413)
Supplement: Supplementary file 1 — Supporting Information [file CTM2-11-e413-s001.docx]

**A consistent arrhythmogenic trait in Brugada syndrome cellular phenotype**

Zeina R Al Sayed et al.

**SUPPLEMENTAL MATERIAL**

**Supplemental methods:**

***Ethical Statement***

The study was conducted according to the principles set forth under the Declaration of Helsinki (1989) and European guidelines for clinical and genetic research. Institutional review board approvals of the study were obtained before the initiation of patient enrollment. The study protocol was reviewed and approved by the regional “*comité de protection des personnes*” ethical committee (approval number: 2010-A01358-31). Regarding the patient-derived biological samples, signed informed consent allowing the experiments to be conducted has been received from all individuals. Any related health information was collected in compliance with applicable law/regulation and with any applicable policy of the ethics committee with jurisdiction over the biological sample collection. All biological samples and their related health information have been provided in coded form such that subjects cannot be identified directly. The provisions of French law, article L1110-4 of the *Code de la santé publique*, related to the privacy and confidentiality of information regarding patients, have been observed.

***Study Population and design***

BrS patients were enrolled according to the presence of a BrS ECG pattern (see below) and with a familial history of sudden death or syncope. Diagnosis of BrS was based on criteria from 2013^1^ with the presence of a type-1 BrS ECG pattern, either spontaneous or induced by intravenous injection of class I antiarrhythmic drugs, in at least one right precordial lead (V1 or V2) positioned in the 2nd, 3rd or 4th intercostal space. Type-1 BrS ECG pattern was defined as a J point elevation higher than 0.2 mV, followed by a coved type ST segment elevation and ended with a negative T wave.

***ECG analysis***

Two physicians blinded to the clinical and genetic status reviewed all baseline ECGs. P wave, PQ interval, QRS, QT peak, QTend, QTc duration (corrected by Bazett formula), and Tpeak-Tend interval (TPE, time interval between the peak and the end of the T wave) were measured in D2, V1, V2 and V3. S-wave duration and amplitude were additionally measured in D1. All measurements were performed using Image J software as previously described.^2,3^ TPE in precordial lead, fragmented QRS, early-repolarization pattern and prominent R wave in lead aVR were determined as previously described.^4,5^

***Genetic analysis***

Patients were screened for mutations in genes previously described in cardiomyopathies and inherited arrhythmias.^6,7^ For this, genomic DNA of probands was extracted from peripheral blood lymphocytes by standard protocols. The DNA yields were assessed by measurements using Quant-IT™ dsDNA Assay Kit, Broad Range (Life Technologies, Q33130). The purity of the DNA was assessed by spectrophotometry (OD 260:280 and 260:230 ratios) using a Nanodrop instrument (Thermo Scientific). DNA integrity was assessed by separation in E-Gel® 96 Agarose Gels, 1% (Life Technologies, G700801). For multiplex amplification, we used the HaloPlex™ Target Enrichment System (Agilent Technologies, 1-500 kb, ILMFST, 96 reactions, G9901B, Protocol Version D.2 (November, 2012)). A custom HaloPlex™ design was used enabling high-throughput sequencing of the coding regions (exons ± 10 bp) of 20 genes previously associated with the BrS:^6,7^

| *SCN5A* | *CACNA1C* | *RANGRF* | *KCNE1L* |
| --- | --- | --- | --- |
| *GPD1L* | *CACNB2* | *PKP2* | *KCNJ8* |
| *SCN1B* | *CACNA2D1* | *FGF12* | *ABCC9* |
| *SCN2B* | *KCNE3* | *SCN10A* | *KCNH2* |
| *SCN3B* | *KCND3* | *TRPM4* | *HCN4* |

Target enrichment and sequencing were performed as previously described.^7^ First, 200 ng of gDNA sample were digested in eight different restriction reactions, each containing two restriction enzymes, to create a library of gDNA restriction fragments. These gDNA restriction fragments were hybridized to the HaloPlex probe capture library. Probes were designed to hybridize and circularize targeted DNA fragments. During the hybridization process, Illumina sequencing motifs including index sequences were incorporated into the targeted fragments.

The circularized target DNA biotinylated HaloPlex probe complexes were captured on magnetic streptavidin beads. We proceeded to a ligation reaction of the circularized complexes followed by an elution reaction before PCR amplification. The amplified target DNA was purified using AMPure XP bead (Beckman Coulter, A63881). To validate enrichment of target DNA in each library sample by microfluidics analysis, we used the 2200 TapeStation (Agilent Technologies, G2964AA), with D1K ScreenTape (Agilent Technologies, 5067-5361), and D1K Reagents (Agilent Technologies, 5067-5362). We ensured that the majority of amplicons range from 175 to 625 bp. Finally, we quantified the library together with other libraries by qPCR using the KAPA Library Quantification Kit (Clinisciences, KK4854). Libraries were pooled in an equimolar concentration and DNA was then denatured with NaOH. Finally, the library pool was diluted to a final concentration of 9 pM before proceeding to 100-bp paired-end Illumina sequencing on HiSeq1500.

Raw sequence reads were aligned to the human reference genome (GRCh37) using BWA-MEM (version 0.7.5a) after removing sequences corresponding to Illumina adapters with Cutadapt v1.2. GATK was used for insertions and deletions (indel) realignment and base recalibration, following GATK DNAseq Best Practices. Variants were called for each sample separately using the Genome Analysis Toolkit GATK (UnifiedGenotyper version 2.8) Variants were considered as rare if the frequency was < 0.01% compared with the Gnomad database.^8^

Variants were considered as having a potential functional consequence if they were annotated with one or more of the following SO terms for at least one RefSeq transcript: “transcript_ablation” (SO:0001893), “splice_donor_variant” (SO:0001575), “splice_acceptor_variant” (SO:0001574), “stop_gained” (SO:0001587), “frameshift_variant” (SO:0001589), “stop_lost” (SO:0001578), “initiator_codon_variant” (SO:0001582), “inframe_insertion” (SO:0001821), “inframe_deletion” (SO:0001822), “missense_variant” (SO:0001583), “transcript_amplification” (SO:0001889). Loss-of-function variants (nonsense variants, frameshift variants and splice site variants) were defined by the following SO terms: “stop_gained”, “frameshift_variant”, “splice_donor_variant”, or “splice_acceptor_variant”. The potential pathogenicity of variants was determined following the American College of Medical Genetics and Genomics (ACMG) guidelines using the CardioVAI tools from EnGenome.^9^

***Generation and validation of hiPSCs***

BrS5^-^ hiPSCs were previously used in a study revealing the role of a variant in *RRAD*, which encodes RAD GTPase, in BrS.^10^ BrS6^-^ hiPSC line, named iBrS1 in a previous study, was derived from a BrS patient with no defined causative variant.^6^ Four different control hiPSC lines were used, including 2 studied previously.^10,11^ BrS4^-^ cutaneous fibroblasts were reprogrammed using Stemgent mRNA reprogramming kit and pluriton medium. hiPSCs derived from dermal cells harvested from BrS1^+^ and BrS3^-^ as well as blood cells obtained from BrS2^+^ and his unaffected family relative (Non-BrS) were reprogrammed using Sendai virus method (Cytotune reprogramming Kit, Life technologies). Up to three clones of each hiPSC line were selected per patient. HiPSCs were maintained on matrigel-coated plates (0.05 mg/ml, BD Biosciences) with StemMACSTM iPS-Brew XF medium (Miltenyi Biotec). Pluripotency of each hiPSC clone was validated by qRT-PCR, immunostaining, and flow cytometry to verify the expression of endogenous pluripotent factors.

***Validation of SCN5A rare variants and genome integrity***

To validate *SCN5A* rare variants carried by BrS1^+^, BrS2^+^ and Non-BrS, genomic DNA from corresponding hiPSC clones was extracted using NucleoSpin® Tissue kit (MACHEREYNAGEL). Variants were verified by sequencing. Single nucleotide polymorphism (SNP) analysis of all hiPSC clones compared to their parental skin fibroblast cells, was used to confirm genome integrity after reprogramming. DNA was extracted from somatic and hiPSC samples using the QIAGEN QiaAmp kit, according to the manufacturer’s recommendations. The gDNA was quantified using a Nanodrop instrument. 200ng of gDNA were outsourced to Integragen Company (Evry, France) for karyotype analysis using HumanCore-24-v1 SNP arrays. This array contains over 300,000 probes distributed throughout the genome with a median coverage of one probe every 5700 bases. All genomic positions were based on Human Genome Build 37 (hg19). Analysis was performed with GenomeStudio software. Chromosome abnormalities were determined by visual inspection of logR ratios and B-allele frequencies (BAF) values and comparing parental cells with hiPSC-derived samples. LogR ratio, the ratio between observed and expected probe intensity, is informative of copy number variation (CNV, i.e. deletions/duplications), whereas BAF is informative of heterozygosity. SNP data were used to compute CNV. In particular, this type of chips allows detecting loss of heterozygosity (LOH), an important concern for hiPSCs, which is not detectable with classical CGH arrays.

***Differentiation of hiPSCs into cardiomyocytes***

Cardiomyocytes were differentiated from hiPSCs using the established matrix sandwich method.^11^ Briefly, when cells reached 90% confluence, an overlay of Growth Factor Reduced Matrigel (0.033 mg/ml, BD Corning) was added. Differentiation was initiated 24 h later by culturing the cells in RPMI1640 medium (Life Technologies) supplemented with B27 (without insulin, Life Technologies), 2 mM L-glutamine (Life Technologies), 1% NEAA (Life Technologies), 100 ng/mL Activin A (Miltenyi), and 10 ng/mL FGF2 for 24 hours. On the next day, the medium was replaced by RPMI1640 medium supplemented with B27 without insulin, 2 mM L-glutamine, 1% NEAA, 10 ng/mL BMP4 (Miltenyi), and 5 ng/mL FGF2 for 4 days. By day 5, cells were cultured in RPMI1640 medium supplemented with B27 complete (Life Technologies), 2 mM L-glutamine and 1% NEAA and changed every two days. Presence of beating cells was considered as the primary hallmark of a successful cardiomyocyte differentiation.

***Transcript expression analysis***

**Quantitative RT-PCR:** Total RNA samples were isolated using the NucleoSpin RNA kit (MACHEREY-NAGEL). One μg of tRNA was reverse transcribed using High-Capacity cDNA Reverse Transcription Kit (Applied Biosystems) following the manufacturer’s instructions. PCR amplification was performed using FAM labeled-TaqMan probes (Applied Biosystems) to verify the pluripotency of derived hiPSCs as compared to parental fibroblasts (OCT3/4 and NANOG). According to the ΔΔCt method, all data were normalized to ACTB and represented relative to controls.

**TaqMan Low Density array (TLDA):** TLDA studies were conducted using beating clusters of hiPSC-CMs obtained from 12 samples of Ctrl, 4 samples of Non-BrS and 14 samples of BrS hiPSC-CMs. One μg of RNA was reverse transcribed into cDNA using SuperScript IV Vilo Master Mix (Thermo Fisher Scientific). TLDA probe selection covered gene families implicated in cardiac ion channel expression and regulation, and cardiomyocyte structure (Table S3). Genes with average Ct > 32 in all compared groups were considered undetectable and excluded from the analysis (SCN10A and ABCC8). Average Ct of remaining genes for each sample was used for data normalization.^12-14^

**3’ Sequencing RNA Profiling (3’SRP):** 3’SRP protocol was performed according to Kilens et al.^15^ Briefly, the libraries were prepared from 10 ng of total RNA. RNA samples were extracted from 26 BrS and 13 control hiPSC samples (a duplicate for each clone obtained at different cell passages) as well as their corresponding differentiated hiPSC-CMs. The mRNA poly(A) tail was tagged with universal adapters, well-specific barcodes and unique molecular identifiers (UMIs) during template-switching reverse transcriptase. Barcoded cDNAs from multiple samples were then pooled, amplified and tagmented using a transposon-fragmentation approach which enriches for 3’ends of cDNA. A library of 350-800 bp was run on an Illumina HiSeq 2500 using a HiSeq Rapid SBS Kit v2 (50 cycles; FC-402-4022) and a HiSeq Rapid PE Cluster Kit v2 (PE-402-4002). Read pairs used for analysis matched the following criteria: all sixteen bases of the first read had quality scores of at least 10 and the first six bases correspond exactly to a designed well-specific barcode. The second reads were aligned to RefSeq human mRNA sequences (hg19) using bwa version 0.7.17. Reads mapping to several transcripts of different genes or containing more than 3 mismatches with the reference sequences were filtered out from the analysis. Digital gene expression profiles were generated by counting the number of UMIs associated with each RefSeq genes, for each sample. R package DESeq2 (Bioconductor) was used to normalize gene expression, and detect differentially expressed genes. Sample correlation matrix was performed using R package ComplexHeatmap (Bioconductor). Cluster 3.0 software was used to perform the differentially expressed gene heatmap. Gene Set Enrichment Analysis (GSEA) was performed using GeneTrail2. Statistically significant categories within the GO Molecular Process were identified using Kolomogorov-Smirnov test and p values were adjusted using the Benjamini and Hochberg method.

***Protein expression analysis***

Beating clusters of hiPSC-CMs were lysed in buffer composed of 1% TritonX-100, 100 mM NaCl, 50 mM Tris-HCl, 1 mM EGTA, 1 mM Na3VO4, 50 mM NaF, 1 mM PMSF and protease inhibitors cocktail (P8340, Sigma-Aldrish). Protein quantification was then conducted using PierceTM BCA Protein Assay Kit (Thermo Fisher). The lysates were denatured for 5 min at 65°C in a mixture of NuPAGE® Sample Reducing Agent (10X) and NuPAGE® LDS Sample Buffer (4X). 40 μg of each sample were loaded onto 12% precast polyacrylamide gels (Bio-Rad). After migration, the proteins were transferred onto Trans-Blot® TurboTM Nitrocellulose Transfer Packs (Bio-Rad). Membranes were saturated with 5% non-fat milk, then incubated with primary antibody (Anti-Na_v_1.5: 14421S Cell Signaling; Anti-Cav1.2: AB5156 Sigma-Aldrich; Anti-TFRC:13-6890 Thermofisher) overnight followed by another incubation for 1 h with an adequate Horseradish peroxidase (HRP)-conjugated secondary antibody. Protein bands were detected using ECL detection system (Bio-Rad) and quantified using Image Lab software.

***Immunostainings***

To validate hiPSC pluripotency, hiPSC-CM differentiation into cardiac lineage as well as COS-7 cell expression, cells were dissociated and seeded onto 8-wells ibidi plates (Biovalley) coated with Matrigel (Corning). After 12 days following hiPSC-CM dissociation, and 24 h following COS-7 transfection, cultured cells were fixed with 4% paraformaldehyde, permeabilized with 0.5% Triton X100 and blocked with 1% BSA. Immunofluorescent stainings were performed using appropriate primary antibodies (Anti-TRA1-60: 14-8863-80 eBioscience™; Anti-OCT4: 14-5841-80 eBioscience™; Anti-troponin I: sc-15368 Santa Cruz; Anti-Na_v_1.5: 14421S Cell Signaling) and their corresponding Alexa conjugated antibodies (Molecular Probes). DAPI was used for nuclear staining. Immunostainings were examined using an inverted epifluorescent microscope (Zeiss Axiovert 200M).

***p.N1722D Na_v_1.5 study in COS-7 cells***

The point mutation p.N1722D (c.1859G>A) in *SCN5A* was introduced using mutated oligonucleotide extension (QuikChange II XL Site-Directed Mutagenesis Kit) in *SCN5A* isoform 2 cDNA (GenBank Acc. Nb. NM_000335). The resulting plasmid was verified by complete sequencing of the cDNA insert. African green monkey kidney-derived cells COS-7, were transiently transfected with 0.4 μg *SCN5A*-expressing plasmid (wild type or mutant) together with 0.4 μg of Navβ1 subunit (SCN1B) plasmid for a 35-mm Petri dish. Transfections were performed using 4 μL JetPEI reagent (Polyplus Transfections, France) according to the manufacturer’s instructions. Enhanced green fluorescent protein (eGFP)-encoding plasmid (1.2 μg) was included to identify transfected cells. One day after transfection, cells were re-plated onto 35-mm Petri dishes for patch clamp experiments.

***Electrophysiological assessment***

**Sodium current (I_Na_) recordings in transfected COS-7 cells.** Currents were recorded 2 days after transfection. Cells were superfused with a solution containing the following (in mM): 145 NaCl, 4 CsCl, 1 CaCl2, 1 MgCl2, 5 HEPES, and 5 glucose, pH=7.4 with NaOH. Patch pipettes were fabricated from borosilicate glass capillaries and had resistances between 1.5 and 2 MΩ when filled with pipette solution (in mM): 90 KCl, 45 K-Gluconate, 10 NaCl and 10 HEPES, pH=7.2 with CsOH. All recordings were made at room temperature (20°C-22°C), after capacitance and series resistance compensation, using an Axopatch 200B amplifier controlled by Axon pClamp 10.6 software through an A/D converter (Digidata 1440A). Data were analyzed using Clampfit 10.6 software (all Molecular Devices).

**Current- and voltage-clamp in hiPSC-CMs.** Cardiomyocytes were dispersed as single cells around day 20 of differentiation, for 20 min in collagenase II (200 U/mL; Gibco) at 37°C. Ten to twelve days after dissociation, spontaneously beating cells were used for patch-clamp recordings. All experiments were conducted at 37°C. Data were collected from a minimum of 3 independent differentiations.

**Action potential (AP) recordings.** Using amphotericin-B perforated-patch configuration, APs were acquired with the same amplifier and converter as above in hiPSC-CMs cells bathed in a Tyrode solution containing (in mM): 140 NaCl, 4 KCl, 1 CaCl2, 0.5 MgCl2, 10 glucose, 10 HEPES; pH 7.4 (NaOH). Borosilicate glass pipettes (2-3 MΩ of tip resistance) were filled with a solution containing (in mM): 125 K-Gluconate, 20 KCl, 5 NaCl, 5 HEPES; pH 7.2 (KOH) and 0.22 amphotericin-B. We first recorded spontaneous APs in order to determine, for each individual included in the study, the proportion of nodal-like, atrial-like and ventricular-like, as previously described.^11^ Then to overcome limited I_K1_ contribution during hiPSC-CM AP, artificial I_K1_ was injected using dynamic patch-clamp.^16^ Both cell stimulation and I_K1_ injection were realized using a custom-made software running on RT-Linux and an A/D converter (National Instrument PCI-6221) connected to the current command of the amplifier. The AP parameters measured were the maximum diastolic potential (MDP), the maximum upstroke velocity of phase 0 depolarization (dV/dt_max_), the AP amplitude and the AP duration at different levels of full repolarization, from 30% (APD30) to 90% (APD90), as previously described.^11^ Following I_K1_ injection, at a peak outward density that sets the membrane potential between -80 to -85mV for all cells, action potential classification into ventricular type was assessed by (APD30-APD40)/(APD70-APD80)>1.45 reflecting the presence of a plateau phase. Cells were paced with a 1-ms 30-35 pA/pF stimulation pulse at 700 ms of cycle length. BrS being a ventricular arrhythmic disease, the analyses were focused on ventricular-like AP. Data from 7 consecutive APs were averaged. GS-458967 (Gilead Sciences) specifically inhibiting I_Na,L_, was solubilized in DMSO and used at 300 nM during AP recording in a solution containing (in mM): 140 NaCl, 4 KCl, 1 CaCl2, 0.5 MgCl2, 30 mannitol, 10 HEPES; pH 7.4 (NaOH).

**Current recordings.** I_Na_ and I_Ca,L_ measurements were recorded in the ruptured-patch configuration and low-pass filtered at, respectively, 10 KHz and 3 KHz using a VE-2 amplifier (Alembic Instrument, Qc, Canada). Cells were bathed using a Tyrode solution containing (in mM): 130 NaCl, 10 CsCl, 1.8 CaCl2, 1.2 MgCl2, 11 glucose and 5 HEPES; pH 7.4 (NaOH). Holding potentials were set to -80 mV and -100 mV, respectively. The series resistance was compensated. Current densities and gating properties were measured using appropriate voltage protocols shown in the relevant figures. After leak subtraction, current densities were calculated by dividing current amplitude by membrane capacitance. Voltage-dependence of activation and inactivation curves were fitted with a Boltzmann function (y=[1+exp{-(V-V1/2)/K}]-1), where V1/2 is the half-maximal voltage of (in)activation and K is the slope factor.

For transient **sodium current (I_Na_)** recording, a local gravity microperfusion system allowed application of an extracellular solution containing (in mM): 20 NaCl, 110 CsCl, 1.8 CoCl2, 1.2 MgCl2, 30 mannitol and 5.0 HEPES; pH 7.4 (CsOH). The pipette solution contained (in mM): 3 NaCl, 133 CsCl, 2 MgCl2, 2 Na2ATP, 2 TEACl, 10 EGTA, 5 HEPES; pH 7.2 (CsOH).

**Late sodium current (I_Na,L_)** was measured as a TTX-sensitive current (Tetrodotoxin Citrate, TOCRIS Bioscience) using an ascending voltage-ramp protocol. The same pipette solution as for peak I_Na_ recording was used. The extracellular solution used in the local gravity microperfusion system had the following composition (in mM): 130 NaCl, 10 CsCl, 1.8 CoCl2, 1 MgCl2, 30 mannitol, 10 HEPES; pH 7.4 (CsOH). TTX was used at a concentration of 0.03 mM.

**Calcium current (I_Ca,L_)** was recorded from cells perfused an extracellular solution containing (in mM): 160 TEACl, 5 CaCl2, 1 MgCl2, 1 MgCl2, 20 mannitol, 10 HEPES, and 0.01 TTX; pH 7.4 (CsOH). The pipette solution contained (in mM): 5 NaCl, 145 CsCl, 2 CaCl2, 5 EGTA, 5 MgATP, 10 HEPES; pH 7.2 (CsOH).

***Mathematical electrogram modeling***

Right-ventricle electrogram was calculated as in Gima and Rudy,^17^ by simulating a heterogeneous transmural wedge (right ventricular outflow tract, 1 Hz, 500th beat shown). This model aims at mimicking the global electrical activity of a row of 165 subendocardial, midmyocardial and subepicardial human ventricular cells.^18,19^ In the original model used by Gima and Rudy, a slow inactivation gate (j) of the Na+ channel was added to include the property of slow recovery,^20^ without modifying fast inactivation (realized with h gate). A few modifications were operated. Since this ‘j’ inactivation was originally as fast as ‘h’ inactivation, it prevents the appearance of a persistent current when altering the ‘h’ gate. So we slowed down this ‘j’ inactivation, but left the kinetics of recovery intact, by modifying the following equation:

If V >= -50 beta_j=0.035*exp(+0.008*p->v)/(1.0+exp(-0.048*(p->v+77.329)).

Thus, in the modified model, any incomplete fast inactivation (for instance, h parameter varies between 1 and 0.166 for BrS2^+^, instead of 1 and zero for Ctrl) gives rise to a late Na^+^ current similar to that observed in Figure 3C (see also Figure below) resulting in the following equation.

*
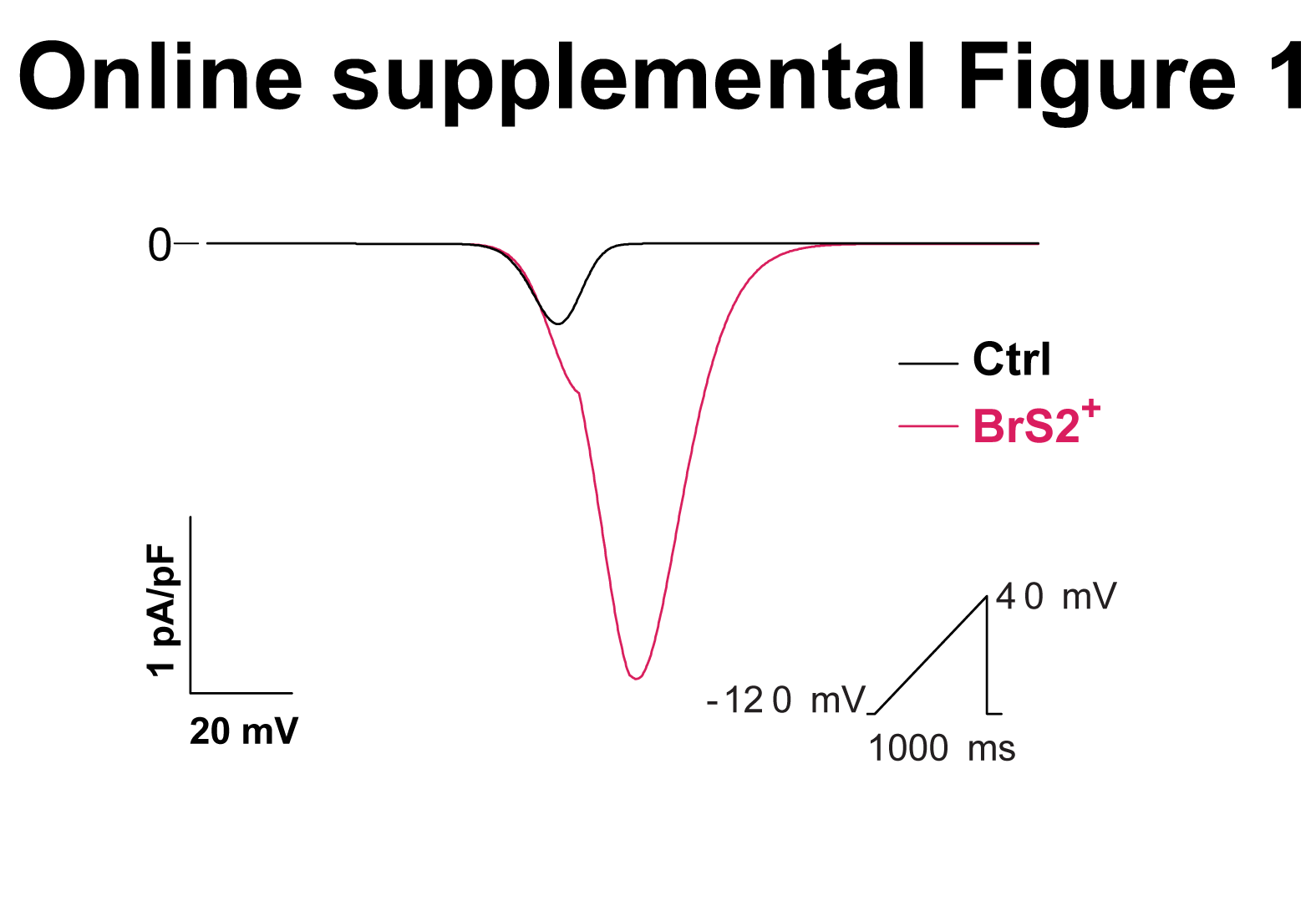
*

*Impact of incomplete inactivation on late sodium current in a simulated Ramp protocol.* Ctrl: h parameter varies between 1 and 0.^17^ BrS: h varies between 1 and 0.116. The ratio of maximal I_Na,L_ current in BrS/Ctrl is 5.4, as in Figure 3C for BrS2^+^.

Noteworthy, this modification does not change inactivation properties of the Na^+^ current in the WT model because fast inactivation (h) is not modified.

Since only a subset of hiPSC-CMs present the late Na^+^ current, we performed simulations with the late Na^+^ current only in midmyocardial cells, giving rise to a J point elevation followed by coved ST segment.^18^

Since decreasing I_Na_ by as much as 73-75% (BrS2^+^ and Non-BrS) was preventing conduction in the Gima and Rudy model, a slighter 30% decrease in I_Na_ was applied.

***Statistical analysis***

Results are expressed as mean ± SEM. Comparisons were made by use of Mann-Whitney test, Student *t*-test, or two-way ANOVA with Bonferroni post-hoc test for repeated measures. Correlations were investigated based on correlation coefficient r_s_ of Spearman.

Values of p < 0.05 were considered statistically significant. Statistical analyses were performed with GraphPad Prism software.

**Supplemental results:**

**Patient characteristics**

Six patients affected by type I BrS (BrS1-6) with a familial history of SCD or syncope were selected, among which two carrying *SCN5A* variants (marked with a ^+^ symbol in BrS1^+^ and BrS2^+^).

Patient #1 (BrS1^+^) was a 35-year-old male with a history of recurrent near-syncope. He presented with a spontaneous BrS ECG pattern. After induction of ventricular fibrillations (VF) during electrophysiological study, an implantable cardioverter-defibrillator (ICD) was implanted with no recurrence of syncope or VF during a 14-year follow-up. Five of his relatives also exhibited a BrS ECG pattern.

Patient #2 (BrS2^+^) was an asymptomatic 55-year-old male whose brother died suddenly at age 38. He presented a spontaneous BrS ECG pattern with occurrence of VF during an electrophysiological study. An ICD was implanted with no occurrence of syncope or VF during a 13-year follow-up. Six of his relatives presented with a BrS ECG pattern including his son who exhibited one spontaneous episode of VF.

Patient #3 (BrS3^-^) was a 35-year-old male who suffered from syncope at night and presented with a spontaneous BrS ECG pattern with VF occurrence during an electrophysiological study. Despite hydroquinidine administration, several episodes of VF were reported. Endocardial catheter ablation of premature ventricular beats was performed without further recurrences under hydroquinidine therapy. Two of his relatives presented with a BrS ECG pattern.

Patient #4 (BrS4^-^) was a 44-year-old male who presented episodes of unexplained syncope at rest. Ajmaline test revealed a BrS ECG pattern. Electrophysiological study did not induce any arrhythmia. Due to familial history of SCD and recurrent syncope, an ICD was implanted without recurrence of syncope or VF during a 13-year follow-up. Type-1 BrS ECG was identified in 8 additional family members.

Patient #5 (BrS5^-^) was a 41-year-old male, previously described by Belbachir *et al*,^10^ who presented recurrent near-syncopes with palpitations and spontaneous BrS ECG pattern. Electrophysiological study induced VF. An ICD was implanted with no recurrence of syncope or VF during a 16-year follow-up. Familial screening identified 6 relatives with a BrS ECG pattern and one with unexplained SCD at age 41.

Patient #6 (BrS6^-^) was a 42-year-old male, previously described by Veerman *et al*.^6^ While he had a spontaneous BrS ECG pattern, he presented an unexplained syncope, and suffered from an out-of-hospital cardiac arrest at night. Three of his relatives presented with a BrS phenotype after ajmaline administration.

An additional Patient #7, not affected by Brugada syndrome (Non-BrS), was recruited. He was a 67-year-old male diagnosed with progressive cardiac conduction defect (PCCD). He was the nephew of patient BrS2^+^. His ECG displayed broad PR interval and prolonged QRS duration, however, no ST segment elevation was detected even after challenge with flecainide.

Four control subjects were also included. One subject who was a relative of BrS5^-^ patient,^10^ in whom BrS was excluded after sodium channel blocker challenge and three other unrelated healthy extra familial controls, including one previously described^11^ and 2 others with different ethnicity.^21^

Representative patient ECGs are presented in Figure S1. Description of patients and their corresponding ECG measurements are depicted in Table S1 and Table S2 respectively.

**Genetic characterization**

Genetic screening of the coding regions of 20 genes, selected according to the American College of Medical Genetics and Genomics (ACMG) guidelines, revealed *SCN5A* pathogenic variants in only two of the six BrS patients. BrS1^+^ carried a c.5164A>G missense rare variant, resulting in asparagine to aspartic acid substitution at position 1722 in the extracellular connecting loop, between segment 5 and 6 in domain IV forming the pore region of Na_v_1.5 (p.N1722D). BrS2^+^ and his nephew (Non-BrS) affected by PCCD carried a 10bp duplication (c.1983–1993dup) in *SCN5A* creating a stop codon (p.A665G-fsX16). Both these variants had not been functionally investigated yet. BrS5^-^ patient presented a previously described rare genetic variant in the *RRAD* gene (p.R211H)^10^ whereas no genetic variation was identified in BrS candidate genes in BrS3^-^, BrS4^-^ or BrS6^-^ patients (Table S1).

**Generation and characterization of hiPSCs and hiPSC-CMs**

Somatic cells were obtained from all studied subjects and were reprogrammed into corresponding hiPSC lines. BrS5^-^, BrS6^-^ and the control hiPSC lines have been previously characterized.^6,10,11,21^ For each of the other 5 newly generated hiPSC lines (BrS1 to 4 and Non-BrS), up to three independent clones were amplified and characterized. SNP analysis verified that control and mutated hiPSC lines were free from any genomic aberrations compared to parental somatic cells (data not shown). The expression of the pluripotent stem cell markers was verified (Figure 1A, 1B and 1C). Genetic screening confirmed that the lines arising from patients carrying *SCN5A* genetic variants, BrS1^+^, BrS2^+^ and Non-BrS harbored the corresponding heterozygous rare variant (Figure 1D).

hiPSC differentiation process into cardiomyocytes was also validated at the transcriptional level. 3’SRP-based global transcriptomic analysis of control and BrS hiPSCs and hiPSC-CMs showed that cells clustered based on their stage (hiPSCs on one side and hiPSC-CMs on the other side) and independently from their genetic background (Figure 1E). Correlation analysis also showed that samples correlated according to their stage, with all hiPSC samples being correlated to other hiPSC samples and similarly for hiPSC-CM samples (Figure 1F). Finally, both Ctrl and BrS hiPSC-CMs presented a comparable global change in gene expression as compared to hiPSC stage (Figure 1G). Immunostaining analysis showed that striated troponin I, a cardiac and muscular specific cytoskeletal protein, was similarly present in all hiPSC-CM lines (Figure 1H). Spontaneous APs, in absence of I_K1_ injection, were classified based on MDP, dV/dt_max_, AP duration and morphology into nodal-like, atrial-like and ventricular-like, as previously described.^11^ The proportion of each cell type was similar between all hiPSC-CM lines, with the ventricular-like type forming the majority of explored hiPSC-CMs (Figure 1I). Altogether, these data confirmed that Ctrl, BrS, and Non-BrS hiPSCs differentiated similarly into cardiomyocytes, and therefore a comparative electrophysiological analysis could be performed to unveil a potential common cellular phenotypic trait of BrS hiPSC-CMs.

**Supplemental references:**

1. Priori SG, Wilde AA, Horie M, et al. HRS/EHRA/APHRS Expert Consensus Statement on the Diagnosis and Management of Patients with Inherited Primary Arrhythmia Syndromes. *Heart Rhythm*. 2013;10:1932‑63.

2. Therasse D, Sacher F, Babuty D, et al. Value of the sodium-channel blocker challenge in Brugada syndrome. *Int J Cardiol*. 2017;245:178‑80.

3. Therasse D, Probst V, Gourraud J-B. Sodium channel blocker challenge in Brugada syndrome: Role in risk stratification. *Int J Cardiol*. 2018;264:100‑1.

4. Gourraud J-B, Barc J, Thollet A, et al. Brugada syndrome: Diagnosis, risk tratification and management. *Arch Cardiovasc Dis*. 2017;110:188‑95.

5. Berthome P, Tixier R, Briand J, et al. Clinical presentation and follow-up of women affected by Brugada syndrome. *Heart Rhythm.* 2018;16:260-267.

6. Veerman CC, Mengarelli I, Guan K, Stauske M, Barc J, Tan HL, Wilde AAM, Verkerk AO, Bezzina CR. hiPSC-derived cardiomyocytes from Brugada Syndrome patients without identified mutations do not exhibit clear cellular electrophysiological abnormalities. *Sci Rep*. 2016;6:30967.

7. Scouarnec SL, Karakachoff M, Gourraud J-B, Lindenbaum P, Bonnaud S, Portero V, Duboscq-Bidot L, Daumy X, Simonet F, Teusan R, Baron E, Violleau J, Persyn E, Bellanger L, Barc J, Chatel S, Martins R, Mabo P, Sacher F, Haïssaguerre M, Kyndt F, Schmitt S, Bézieau S, Marec HL, Dina C, Schott J-J, Probst V, Redon R. Testing the burden of rare variation in arrhythmia-susceptibility genes provides new insights into molecular diagnosis for Brugada syndrome. *Hum Mol Genet*. 2015;ddv036.

8. Karczewski KJ, Francioli LC, Tiao G, Cummings BB, Alföldi J, Wang Q, Collins RL, Laricchia KM, Ganna A, Birnbaum DP, Gauthier LD, Brand H, Solomonson M, Watts NA, Rhodes D, Singer-Berk M, Seaby EG, Kosmicki JA, Walters RK, Tashman K, Farjoun Y, Banks E, Poterba T, Wang A, Seed C, Whiffin N, Chong JX, Samocha KE, Pierce-Hoffman E, Zappala Z, O’Donnell-Luria AH, Minikel EV, Weisburd B, Lek M, Ware JS, Vittal C, Armean IM, Bergelson L, Cibulskis K, Connolly KM, Covarrubias M, Donnelly S, Ferriera S, Gabriel S, Gentry J, Gupta N, Jeandet T, Kaplan D, Llanwarne C, Munshi R, Novod S, Petrillo N, Roazen D, Ruano-Rubio V, Saltzman A, Schleicher M, Soto J, Tibbetts K, Tolonen C, Wade G, Talkowski ME, Consortium TGAD, Neale BM, Daly MJ, MacArthur DG. Variation across 141,456 human exomes and genomes reveals the spectrum of loss-of-function intolerance across human protein-coding genes. *bioRxiv*. 2019;531210.

9. Richards S, Aziz N, Bale S, Bick D, Das S, Gastier-Foster J, Grody WW, Hegde M, Lyon E, Spector E, Voelkerding K, Rehm HL, ACMG Laboratory Quality Assurance Committee. Standards and guidelines for the interpretation of sequence variants: a joint consensus recommendation of the American College of Medical Genetics and Genomics and the Association for Molecular Pathology. *Genet Med Off J Am Coll Med Genet*. 2015;17:405‑24.

10. Belbachir N, Portero V, Al Sayed ZR, et al. RRAD mutation causes electrical and cytoskeletal defects in cardiomyocytes derived from a familial case of Brugada syndrome. *Eur Heart J*. 2019;ehz308.

11. Es-Salah-Lamoureux Z, Jouni M, Malak OA, et al. HIV-Tat induces a decrease in IKr and IKs via reduction in phosphatidylinositol-(4,5)-bisphosphate availability. *J Mol Cell Cardiol*. 2016;99:1‑13.

12. Wang W-X, Danaher RJ, Miller CS, Berger JR, Nubia VG, Wilfred BS, Neltner JH, Norris CM, Nelson PT. Expression of miR-15/107 family microRNAs in human tissues and cultured rat brain cells. *Genomics Proteomics Bioinformatics*. 2014;12:19‑30.

13. Mestdagh P, Van Vlierberghe P, De Weer A, Muth D, Westermann F, Speleman F, Vandesompele J. A novel and universal method for microRNA RT-qPCR data normalization. *Genome Biol*. 2009;10:R64.

14. Bockmeyer CL, Säuberlich K, Wittig J, Eßer M, Roeder SS, Vester U, Hoyer PF, Agustian PA, Zeuschner P, Amann K, Daniel C, Becker JU. Comparison of different normalization strategies for the analysis of glomerular microRNAs in IgA nephropathy. *Sci Rep*. 2016;6:31992.

15. Kilens S, Meistermann D, Moreno D, et al. Parallel derivation of isogenic human primed and naive induced pluripotent stem cells. *Nat Commun*. 2018;9:360.

16. Meijer van Putten, Mengarelli I, Guan K, et al. Ion channelopathies in human induced pluripotent stem cell derived cardiomyocytes: a dynamic clamp study with virtual IK1. *Front Physiol*. 2015;6:7.

17. Gima K, Rudy Y. Ionic current basis of electrocardiographic waveforms: a model study. *Circ Res.* 2002;90:889‑96.

18. Zygmunt AC, Eddlestone GT, Thomas GP, et al. Larger late sodium conductance in M cells contributes to electrical heterogeneity in canine ventricle. *Am J Physiol Heart Circ Physiol*. 2001;281:H689-697.

19. Li GR, Feng J, Yue L, Carrier M. Transmural heterogeneity of action potentials and Ito1 in myocytes isolated from the human right ventricle. *Am J Physiol*. 1998;275:H369-377.

20. Luo CH, Rudy Y. A model of the ventricular cardiac action potential. Depolarization, repolarization, and their interaction. *Circ Res*. 1991;68:1501‑26.

21. Al Sayed ZR, Canac R, Cimarosti B, Bonnard C, Gourraud JB, Hamamy H, Kayserili H, Girardeau A, Jouni M, Jacob N, Gaignerie A, Chariau C, David L, Forest V, Marionneau C, Charpentier F, Loussouarn G, Lamirault G, Reversade B, Zibara K, Lemarchand P, Gaborit N. Human model of IRX5 mutations reveals key role for this transcription factor in ventricular conduction. *Cardiovasc Res*. 2020;8:cvaa259.

22. Morita H, Kusano KF, Miura D, et al. Fragmented QRS as a marker of conduction abnormality and a predictor of prognosis of Brugada syndrome. *Circulation*. 2008;118:1697-1704.

**Supplemental figures:**

**Figure S1:**


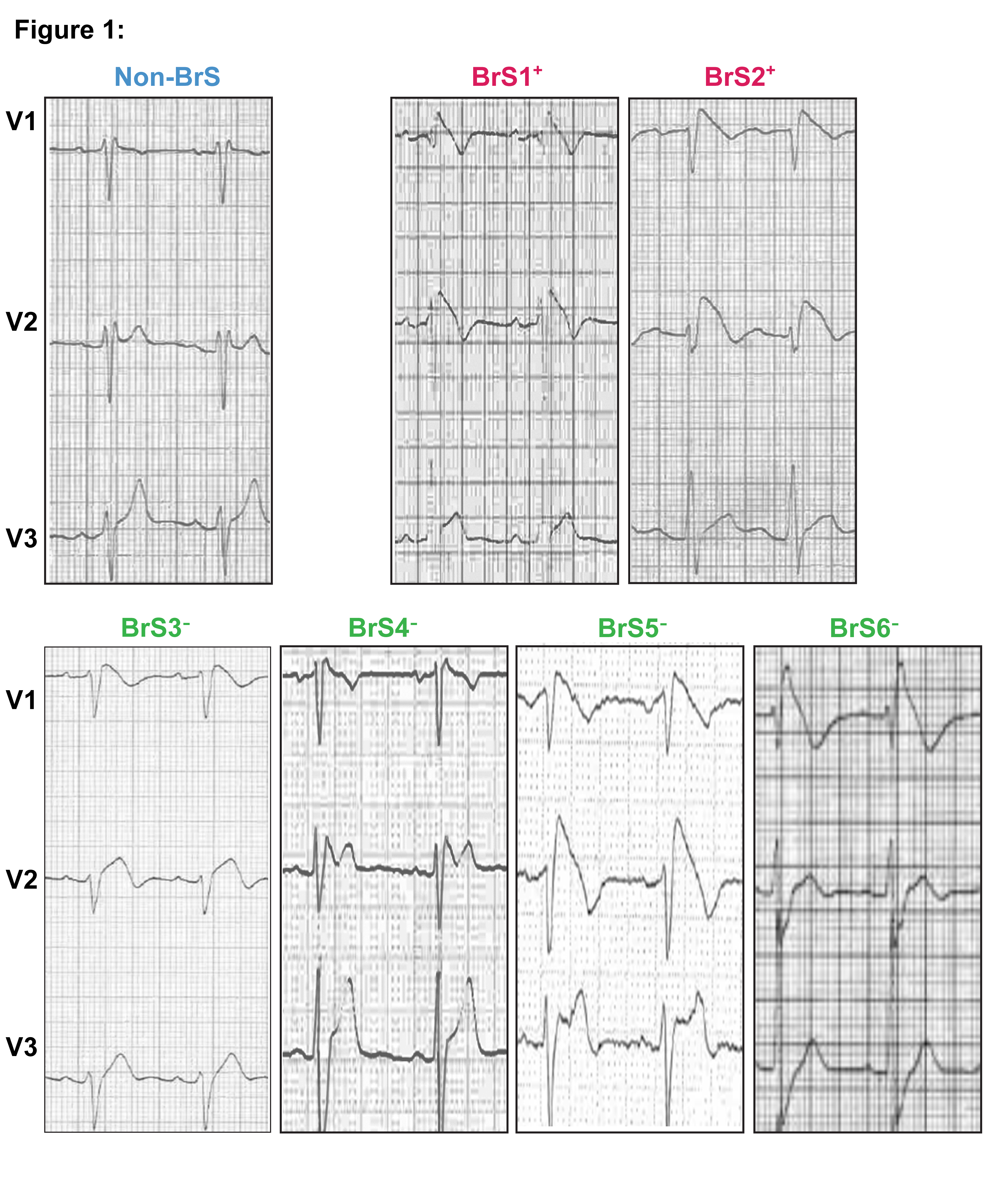


**Figure S1. Electrocardiogram of all subjects from whom hiPSCs were derived**

Leads V1 to V3 from electrocardiogram of Non-BrS and BrS1-6 patients. + and – signs refer to the presence and absence of a *SCN5A* alteration variant, respectively. Type 1 BrS characteristics are visible in all BrS ECG and conduction defect is visible in Non-BrS ECG (right branch block).

**Figure S2:**


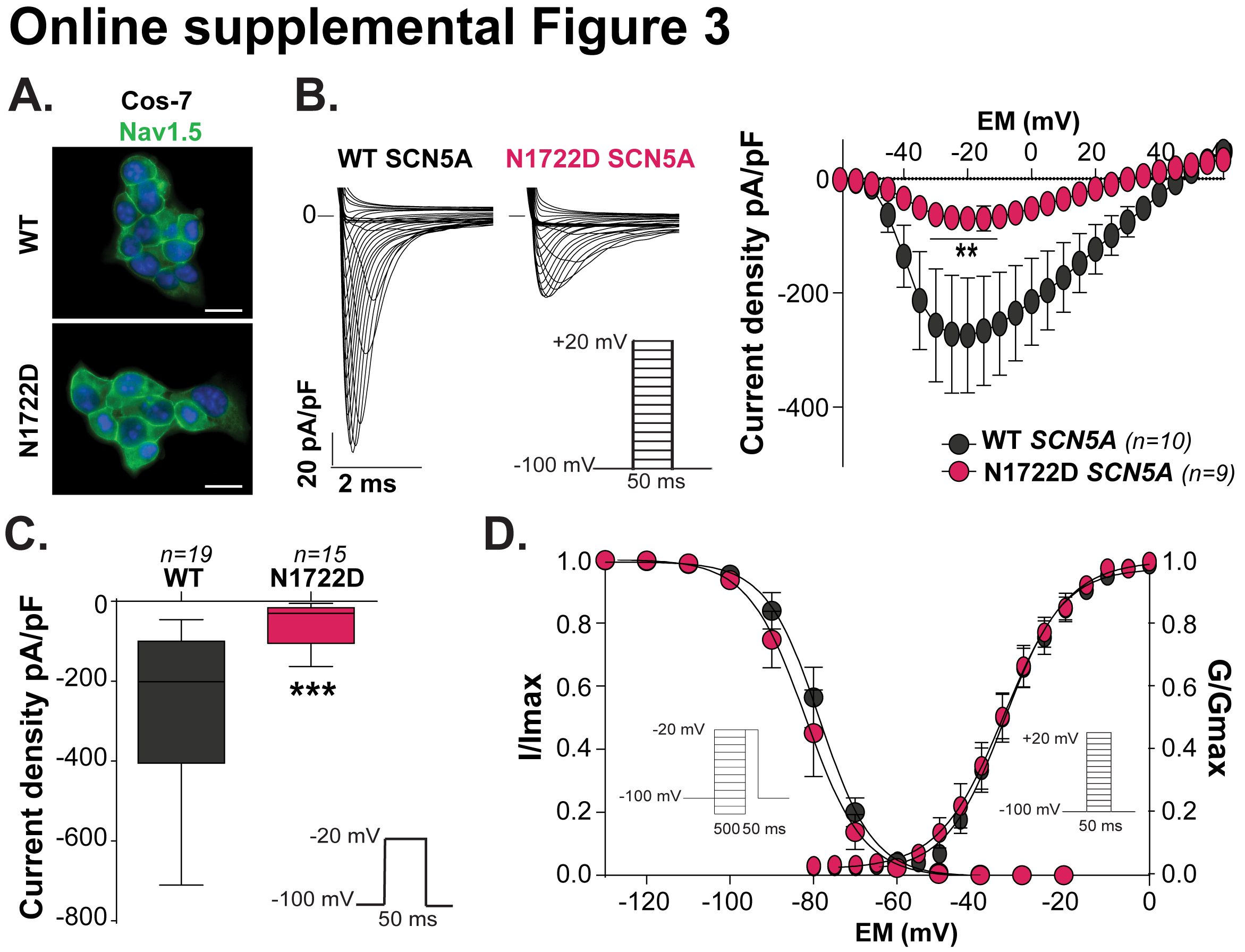


**Figure S2: Electrophysiological characterization of N1722D*-SCN5A*-expressing COS-7 cells.**

The effects of N1722D-*SCN5A* variant present in BrS1^+^ hiPSC-CMs were characterized using COS-7 cells transfected with plasmids expressing either wild type (WT-*SCN5A*) or N1722D-*SCN5A* cDNA.

A. Illustrative immunostainings of Na_v_1.5 (green) in COS-7 cells transfected with WT *SCN5A* and N1722D-*SCN5A* cDNA, showing robust Na_v_1.5 expression of the variant at the cell surface. Nuclei were stained with DAPI (blue). B. Representative whole-cell current recorded in COS-7 cells overexpressing wild type (WT) and N1722D-*SCN5A* cDNA (left panel; voltage protocol in inset). Mean peak I_Na_ current densities (pA/pF) vs. membrane potential (Vm) (right panel). ** p<0.01 vs. WT (Two-way ANOVA with Bonferroni post-hoc test). C. Peak I_Na_ current densities, measured at -20 mV (Tukey plot), unveiling a significant reduction, by about 2 folds, of I_Na_ in N1722D-*SCN5A* transfected-COS-7 cells. *** p<0.001 vs. control (*t*-test).

D. I_Na_ voltage-dependence of inactivation and activation. For inactivation, I_Na_ was normalized to maximum (I/Imax), and plotted as a function of the potential of conditioning pulse that preceded the test pulse to -20 mV (inactivation; inset: voltage protocol). For activation, GNa (i.e. I_Na_/(Vm-E_Na_), E_Na_ being the equilibrium potential for Na+ ions) was normalized to maximum, and plotted as a function of Vm, the potential of the test pulse I_Na_ voltage dependence of activation. GNa (as I_Na_/(Vm-E_Na_), E_Na_ being the equilibrium potential for Na^+^ ions) was normalized to maximum, and plotted as a function of Vm, the potential of the test pulse (activation: same voltage protocol as in B). This analysis did not reveal any modification of steady-state activation and inactivation, as in BrS1^+^ hiPSC-CMs.


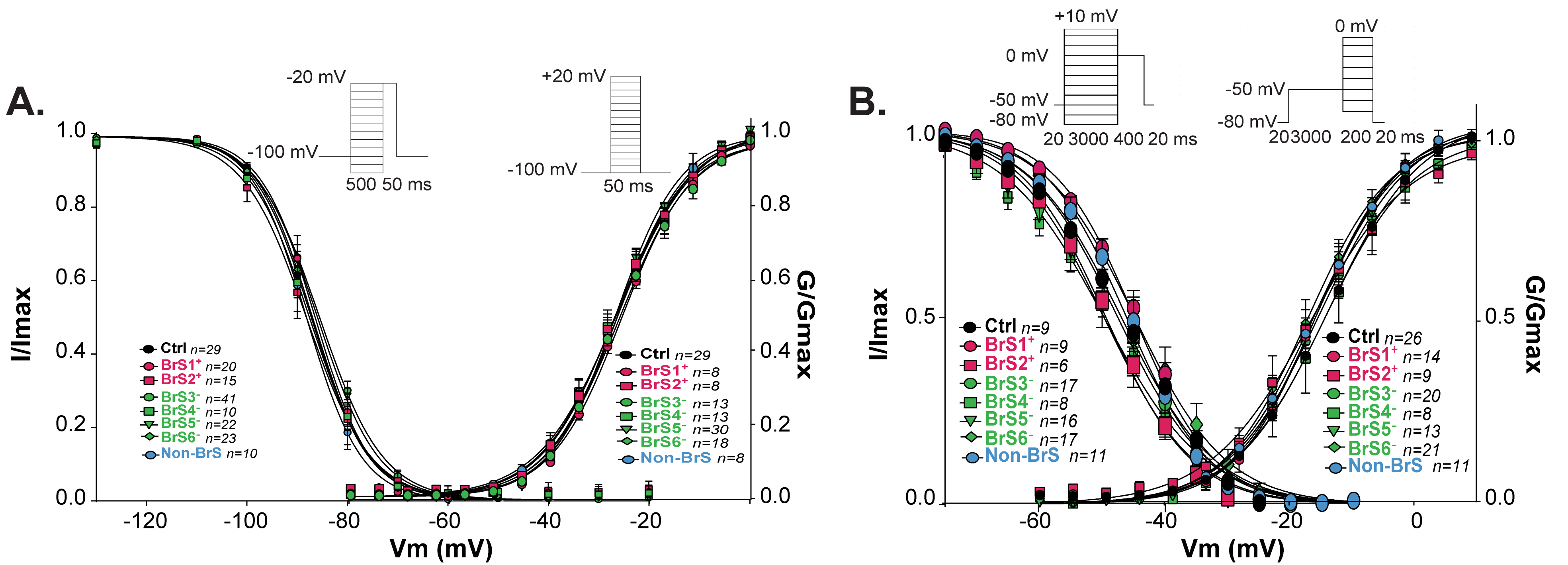
**Figure S3**

**Figure S3. I_Na_ and I_Ca,L_ steady-state activation and inactivation gating properties in** **BrS hiPSC-CMs as compared to controls.**

1. Left: I_Na_ voltage-dependence of inactivation. I_Na_ was normalized to its maximum value, and plotted as a function of the potential of conditioning pulse that preceded the -20-mV test pulse (inset: voltage protocol).

Right: I_Na_ voltage-dependence of activation. GNa (as I_Na_/(Vm-E_Na_), E_Na_ being the equilibrium potential for Na^+^ ions) was normalized to its maximum value, and plotted as a function of the potential of the test pulse (Vm; inset: voltage protocol).

1. I_Ca,L_ voltage-dependence of activation and inactivation (inset: voltage protocol).

**Figure S4:**


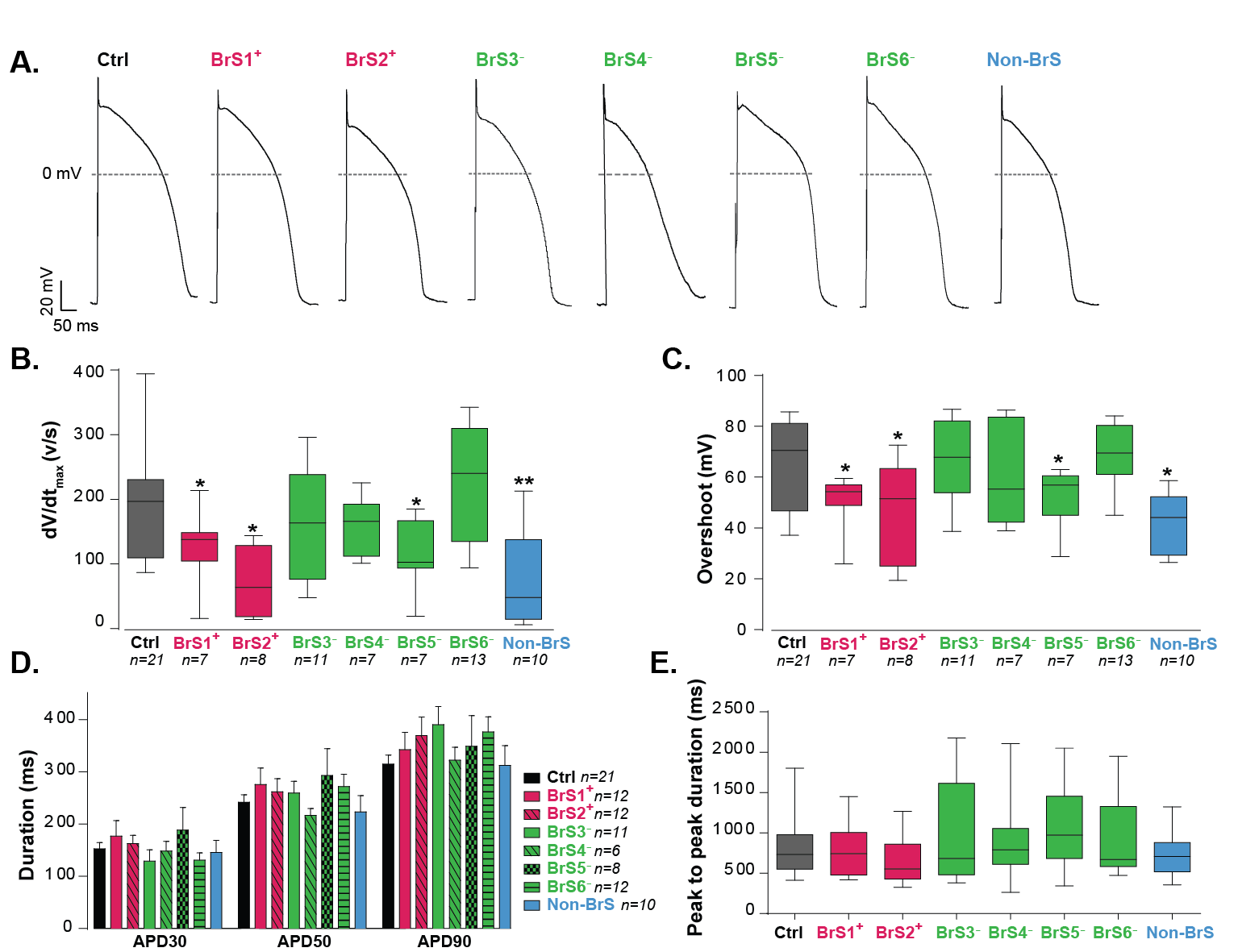


**Figure S4. Ventricular action potential (AP) parameters in** **BrS hiPSC-CMs as compared to controls.**

1. Representative ventricular-like AP when paced at 700 ms cycle length and when artificial I_K1_ was injected (dynamic current-clamp). APs are defined as ventricular-like when (APD_30_-APD_40_)/(APD_70_-APD_80_)>1.45.
2. Maximum upstroke velocity (dV/dt_max_) of ventricular-like APs (Tukey plot). Conditions as in A. *p<0.05 *vs*. control (Mann-Whitney test).
3. AP overshoot from ventricular-like hiPSC-CMs. Conditions as in A. * p<0.05; *vs* control (*t*-test).

The AP maximum upstroke velocity (dV/dt_max_) and overshoot were reduced in hiPSC-CMs presenting a reduction in I_Na_.

1. Ventricular-like AP duration (APD) at 30%, 50% and 90% of full repolarization, showing that consistent with the absence of QT duration modification in BrS patients’ ECGs, no difference in AP duration was observed between BrS and Ctrl hiPSC-CMs. Conditions as in A.
2. Beating frequencies of investigated cell lines. Box plots presenting peak-to-peak durations between action potentials, averaged for all spontaneously recorded action potentials (p = ns; One-way Anova test)

**Figure S5:**


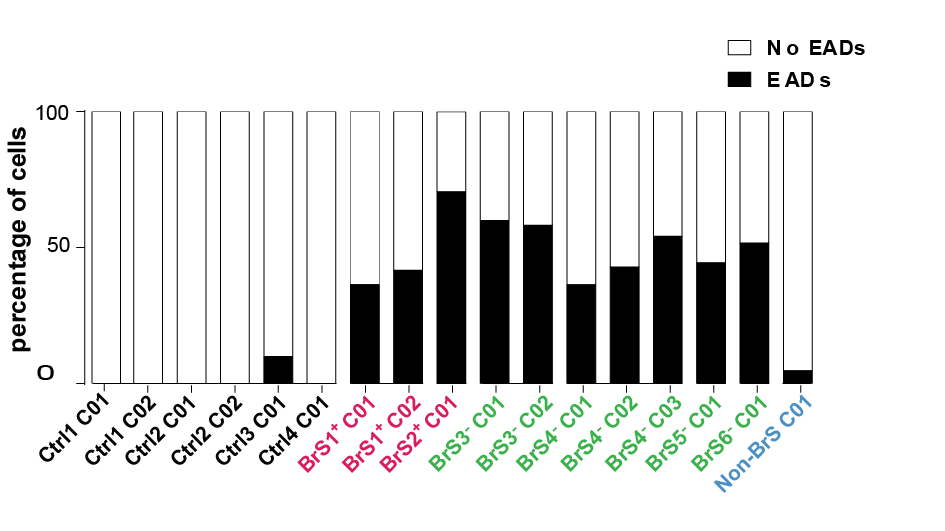


**Figure S5. Proportions of ventricular action potentials with or without EADs.**

Percentage of ventricular-like hiPSC-CMs presenting at least 1 EAD, irrespective of the current-clamp conditions, for each investigated clone of each hiPSC line.

**Supplemental Tables:**

**Table S1.** Patient description


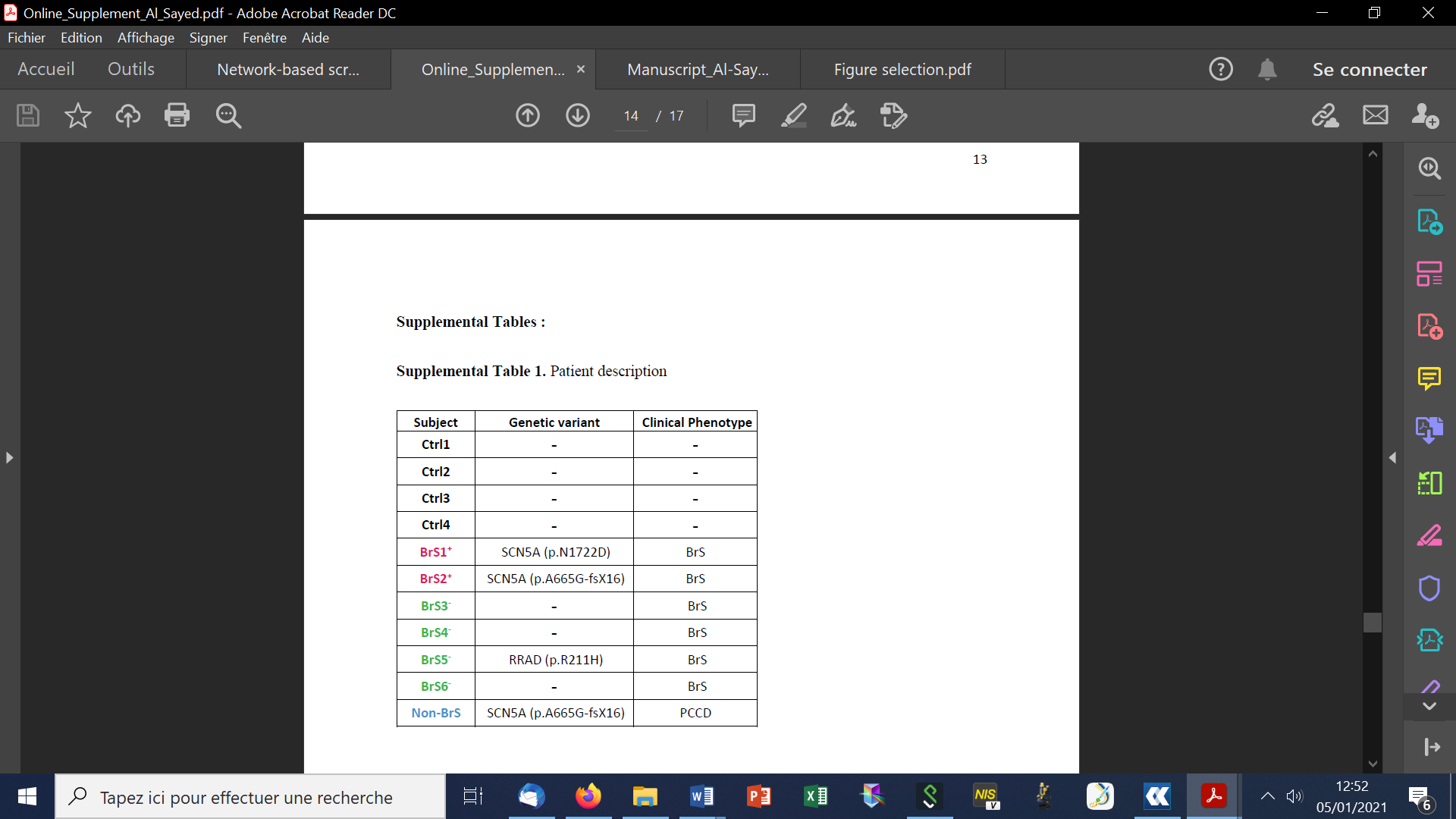


**Table S2.** ECG patient characteristics.

ECGs were performed in leads D1, D2, V1, V2, V3 and recordings used the following parameters: 25 mm/s, 0.1 mV/mm.

RR interval duration (RR); S wave duration and amplitude (S); P wave duration (P); PR interval duration (PR); QRS duration (QRS); QT peak interval (QTp); QT end interval (QTe); Tpeak-to -Tend interval (TPE); J wave amplitude (J); Early Repolarization Pattern (ERP); Fragmented QRS according to Morita H et al.^22^ (Frag); Concave aspect of the ST segment elevation (Concave aspect); Yes (y); No (n).


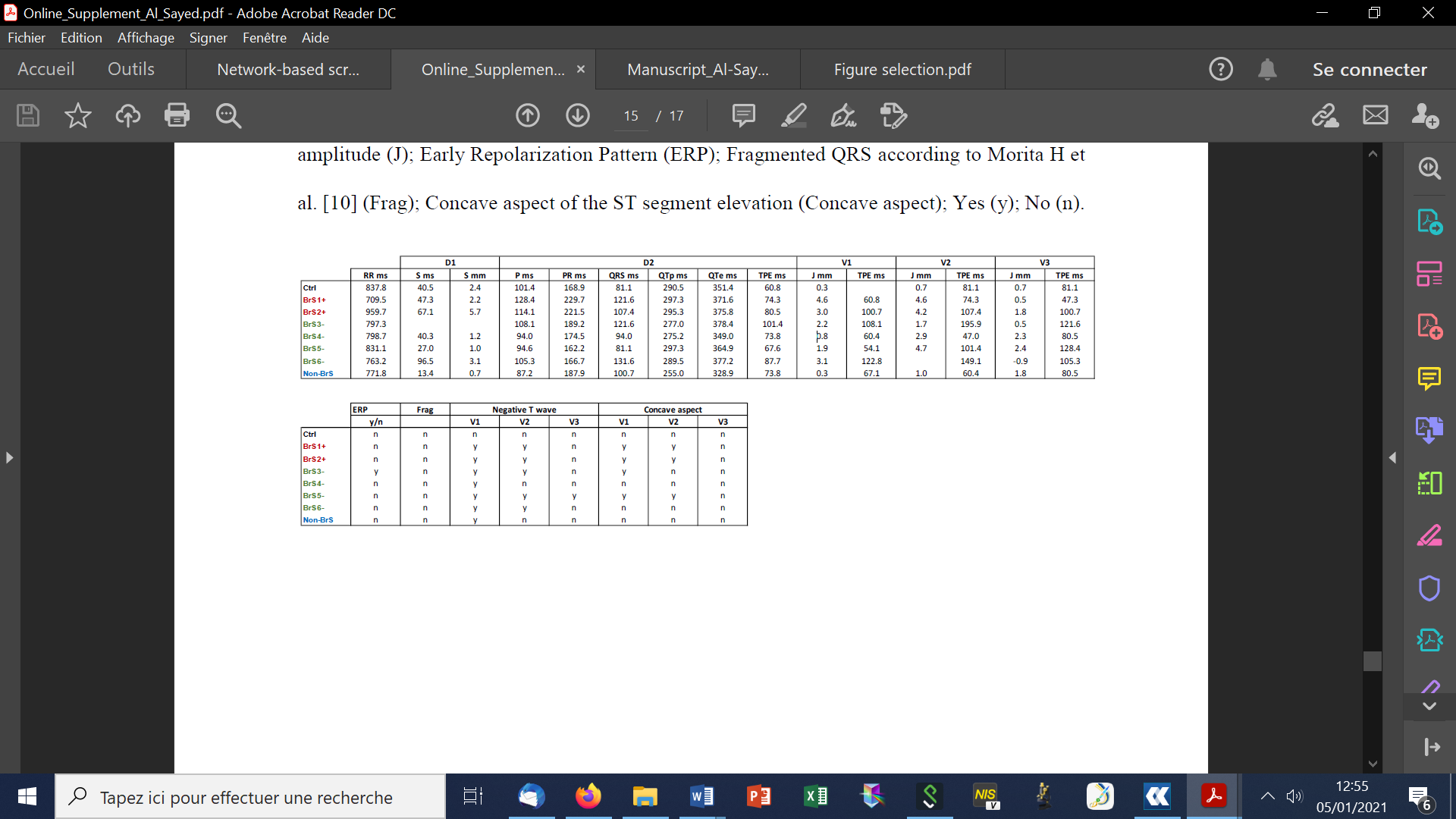

**Table S3.** TLDA probe references and corresponding genes.

**Table S4.** Mean value ± SEM of I_Na_ and I_Ca,L_ activation and inactivation kinetics parameters in the different hiPSC-CMs lines. V1/2 and K represent voltage of half-maximum (in)activation and slope factor, respectively.


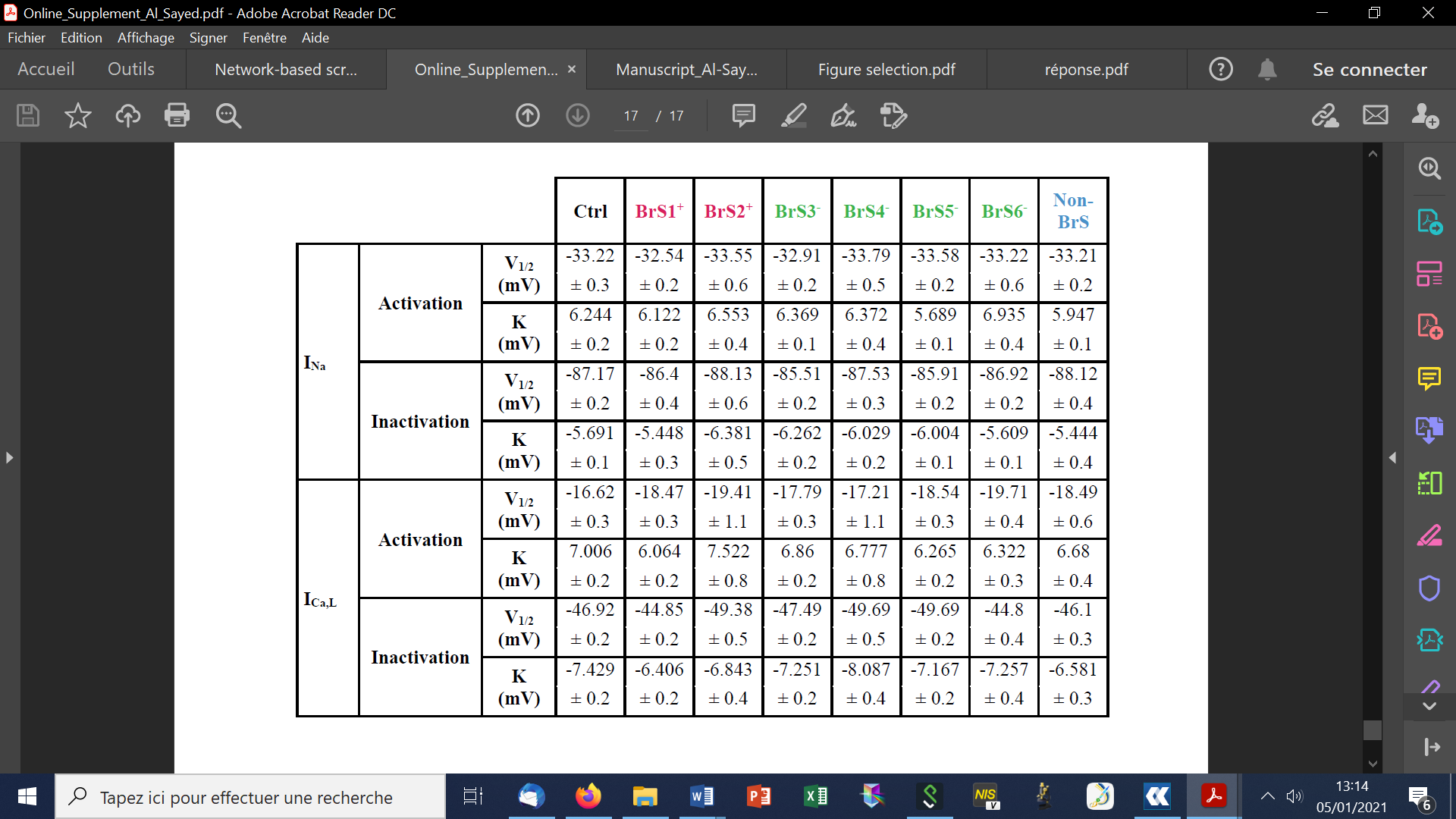


**Table S5.** Correlation between ECG parameters and the corresponding hiPSC-CM sodium currents.

|  |  | **Correlation coefficient r_s_ of Spearman** | | |
| --- | --- | --- | --- | --- |
|  |  | **Late I_Na_** |  | **Peak I_Na_** |
|  | **RR (ms)** | **0.48** |  | **-0.07** |
| **D1** | **S duration (ms)** | **-0.55** |  | **0.00** |
|  | **S amplitude (mm)** | **-0.10** |  | **0.26** |
| **D2** | **P (ms)** | **-0.47** |  | **0.01** |
|  | **PR (ms)** | **-0.63** |  | **0.20** |
|  | **QRS (ms)** | **-0.55** |  | **-0.31** |
|  | **QTp (ms)** | **-0.40** |  | **-0.14** |
|  | **QTe (ms)** | **-0.32** |  | **-0.14** |
|  | **TPE (ms)** | **-0.07** |  | **-0.14** |
| **V1** | **J amplitude (mm)** | **-0.81 *** |  | **0.02** |
|  | **TPE (ms)** | **0.11** |  | **-0.14** |
| **V2** | **J amplitude (mm)** | **-0.86 *** |  | **0.14** |
|  | **TPE (ms)** | **-0.33** |  | **-0.26** |
| **V3** | **J amplitude (mm)** | **0.23** |  | **0.19** |
|  | **TPE (ms)** | **-0.23** |  | **-0.31** |

Each ECG parameter was tested for its correlation with either I_Na,L_ or peak I_Na_ density from the corresponding hiPSC-CMs. While the J point elevation correlated significantly with I_Na,L_ density, it did not correlate with hiPSC-CM peak I_Na_ density. Statistical significance threshold of r_s_ was p < 0.05 (*).
